# Expansion and differentiation of human hepatocyte-derived liver progenitor-like cells and their use for the study of hepatotropic pathogens

**DOI:** 10.1038/s41422-018-0103-x

**Published:** 2018-10-25

**Authors:** Gong-Bo Fu, Wei-Jian Huang, Min Zeng, Xu Zhou, Hong-Ping Wu, Chang-Cheng Liu, Han Wu, Jun Weng, Hong-Dan Zhang, Yong-Chao Cai, Charles Ashton, Min Ding, Dan Tang, Bao-Hua Zhang, Yi Gao, Wei-Feng Yu, Bo Zhai, Zhi-Ying He, Hong-Yang Wang, He-Xin Yan

**Affiliations:** 1 International Cooperation Laboratory on Signal Transduction, Eastern Hepatobiliary Surgery Hospital, Second Military Medical University, Shanghai, China; 2 National Center for Liver Cancer, Shanghai, China; 3 Institute for Regenerative Medicine, Shanghai East Hospital, School of Life Sciences and Technology, Tongji University, Shanghai, China; 4 Department of Hepatobiliary Surgery II, Zhujiang Hospital, Southern Medical University, Guangzhou, China; 5 Celliver Biotechnology Inc., Shanghai, China; 6 Zilkha Neurogenetic Institute, University of Southern California, Los Angeles, CA USA; 7 Department of Interventional Oncology, Renji Hospital, Jiaotong University School of Medicine, Shanghai, China; 8 Department of Anesthesiology and Critical Care Medicine, Renji Hospital, Jiaotong University School of Medicine, Shanghai, China

**Keywords:** Reprogramming, Regeneration

## Abstract

The study of pathophysiological mechanisms in human liver disease has been constrained by the inability to expand primary hepatocytes in vitro while maintaining proliferative capacity and metabolic function. We and others have previously shown that mouse mature hepatocytes can be converted to liver progenitor-like cells in vitro with defined chemical factors. Here we describe a protocol achieving efficient conversion of human primary hepatocytes into liver progenitor-like cells (HepLPCs) through delivery of developmentally relevant cues, including NAD ^+ ^-dependent deacetylase SIRT1 signaling. These HepLPCs could be expanded significantly during in vitro passage. The expanded cells can readily be converted back into metabolically functional hepatocytes in vitro and upon transplantation in vivo. Under three-dimensional culture conditions, differentiated cells generated from HepLPCs regained the ability to support infection or reactivation of hepatitis B virus (HBV). Our work demonstrates the utility of the conversion between hepatocyte and liver progenitor-like cells for studying HBV biology and antiviral therapies. These findings will facilitate the study of liver diseases and regenerative medicine.

## Introduction

The liver may undergo a systemic injurious responses, upon exposure to a diverse set of metabolic, toxic, and inflammatory insults, resulting in global hepatocelluar damage and impaired hepatocyte self-renewal.^1^ Under circumstances of liver injury, a population of liver progenitor-like cells rapidly emerges and extensively expands. The source of these biphenotypic, progenitor-like cells has been unclear until very recently.^2^ It is reported that human and mouse hepatocytes can convert to biliary-like progenitor cells in response to injury which can consequently produce hepatocyte progeny to replenish the inhibited cellular compartment.^3^ Other studies have shown that cholangiocytes can also act as facultative liver stem cells.^4,5^ These findings suggest that both hepatocytes and cholangiocytes might become liver progenitors to rescue hepatocytes during liver injury.^6^

The development of an in vitro experimental setting to generate human liver progenitors either from hepatocytes or from cholangiocytes will be of great importance. It could not only help to improve our understanding of the origin of liver progenitor cells and reprogramming mechanisms but offer an unlimited cell source for generation of functional hepatocytes, which have broad applications in clinical medicine and disease modeling. Recently, we and others have demonstrated that mouse hepatocytes could convert to liver progenitor-like cells in culture,^7,8^ recapitulating the reversible ductal metaplasia for restoration of hepatocyte mass during liver injury.^3^ However, the approach seemed not to be successful in human hepatocyte reprogramming.^7^

Here we report an approach for efficient expansion and differentiation of human hepatocyte-derived liver progenitor-like cells in vitro that relies on active SIRT1 signaling. Such progenitor-like cells can re-differentiate to acquire mature hepatic functions. More importantly, the three-dimensional differentiated cells form spheroids in suspension and express hepatitis B virus (HBV) receptor sodium taurocholate cotransporting polypeptide (NTCP)^9^ at a level similar to primary hepatocytes. This system can be maintained over multiple weeks to recapitulate hepatic life cycles for hepatitis B virus infection and reactivation in vitro. Together, these findings demonstrate that this system serves as a suitable model for the study of host interactions with HBV and anti-viral therapies.

## Results

### Conversion of human primary hepatocytes to liver progenitor-like cells

**Fig. 1:**
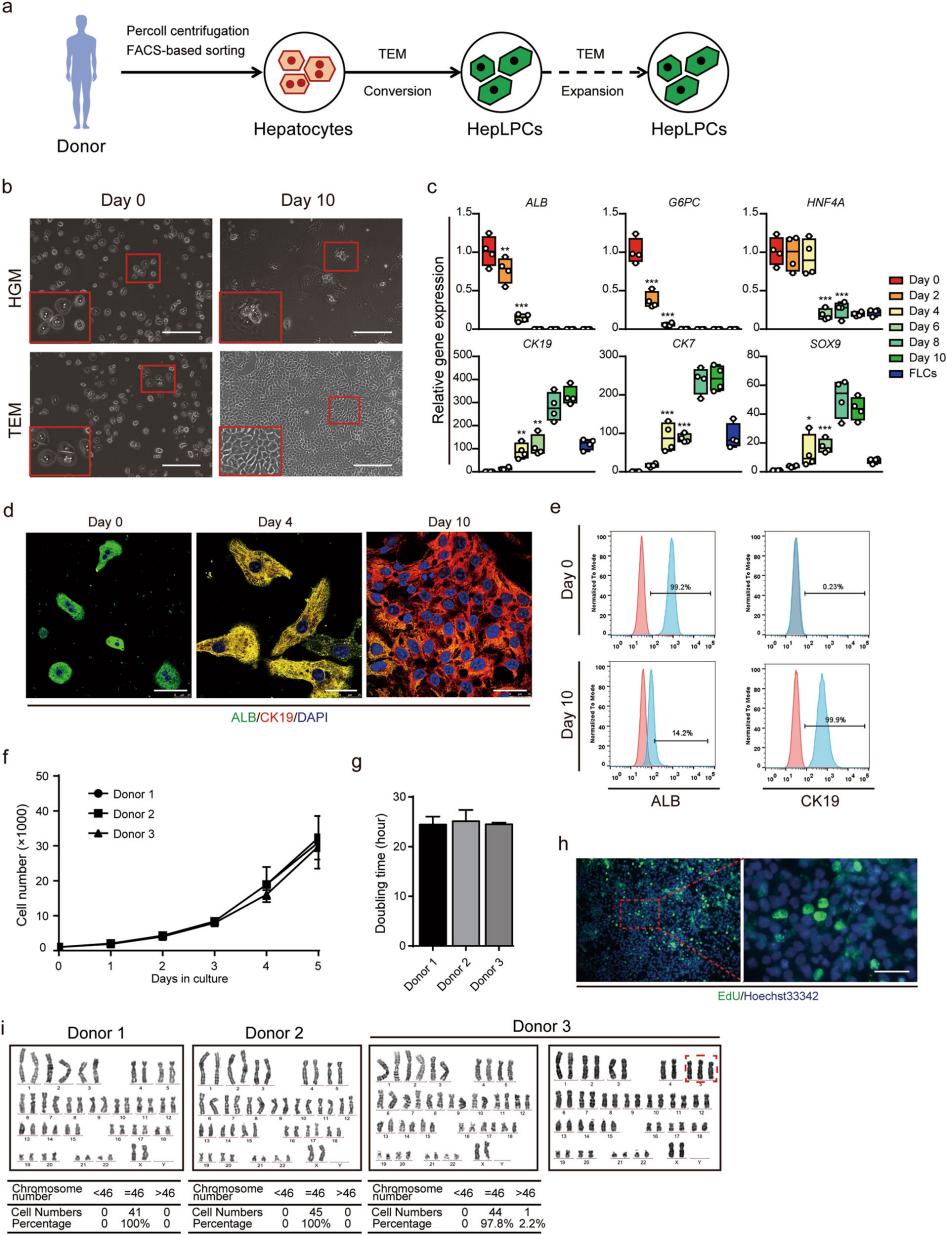
Generation of human hepatocytes-derived liver progenitor-like cells in vitro. **a** Overview of the protocol used to convert PHCs into HepLPCs. **b** Light microscopy images of PHCs cultured in HGM or TEM at day 0 and day 10. Scale bars, 200 µm. **c** QPCR analyses for the expression of the indicated genes during TEM culture from day 0 to day 10. FLCs, fetal liver cells, 5 gestational weeks (one-way ANOVA with Dunnett correction for multiple comparisons, *n* = 4 donors, *P < 0.05, **P < 0.01, ***P < 0.001). **d** Immunofluorescence analyses demonstrating the expression of ALB and CK19. Scale bars, 50 µm. **e** Flow cytometric analysis showing the proportion of ALB or CK19 positive-cells in the populations. Blue, positive-cells; red, negative controls. **f** Growth curves illustrate the number of cells counted per well at different time points. Results expressed as mean ± s.d. of three independent cultures derived from three donors. **g** Doubling time calculated for three donors. Error bars represent s.d.; *n* = 3. **h** Edu incorporation is detected at passage 10. Scale bar, 50 µm. **i** Representative karyotype images of three independently established HepLPCs. Two lines maintained normal diploid karyotypes, whereas the third was partly triploid for Chromosome 5

We have previously identified a transition and expansion medium (TEM) that allows for the conversion of mouse hepatocyte to liver progenitor-like cell (LPC) in vitro.^8^ This led us to ask whether we could use a similar approach to convert human hepatocytes into progenitor cells (Fig. 1a). To test this idea, we first isolated human primary hepatocytes from normal liver tissues using FACS-based sorting to exclude CD24^+^ or EpCAM^+^ progenitor cells (Fig. 1a, Supplementary information, Fig. S11a–c, Table S1). The sorted cells were further validated by ASGPR1 expression (Supplementary information, Fig. S1c). When cultured in TEM, a proportion of purified hepatocytes underwent active division whereas most appeared in stationary phase in HGM during 6-days of culture as assessed by time-lapse imaging (Supplementary information, Movies S1 and S2). The proportion of hepatocytes with greater than four progeny cells during the 6-day culture was 52.2 ± 2.8% under TEM conditions (Supplementary information, Fig. S1d, e). After 10 days of culture, TEM-induced proliferating cells displayed typical features of progenitor cells with a high nucleus/cytoplasm ratio (Fig. 1b). Gene and protein expression levels of multiple hepatic markers decreased while LPC markers increased gradually under the TEM culture conditions, suggesting progressive hepatocyte-to-LPC conversion (Fig. 1c–e). Upon the sequential withdrawal of individual factors from TEM, colony formation efficiency decreased accordingly, suggesting that each factor had a positive influence on the hepatocyte-to-LPC conversion (Supplementary information, Fig. S1f–h). Hepatocyte-derived liver progenitor-like cells (HepLPCs) grew to form a continuous monolayer and the mean population doubling time was approximately 24.7 ± 1.4 h (Fig. 1f, g). Edu incorporation confirmed that HepLPCs maintained their proliferative state to at least passage 10 (Fig. 1h). Karyotypic analyses of three independently established HepLPCs lines showed that two of them maintained normal diploid karyotypes, whereas the third one partly was triploid for Chromosome 5 at passage 10 (Fig. 1i, Supplementary information, Fig. S1i). The derivation of HepLPCs from human primary hepatocytes was further confirmed using a lentivirus-mediated lineage tracing vector carrying TBG-EGFP-T2A-puro, in which GFP and a puromycin resistance gene are driven by a liver-specific promoter (TBG promoter), to label mature human hepatocytes (Supplementary information, Fig. S2a). The converted reporter hepatocytes expressed liver progenitor-related markers at levels similar to those of HepLPCs derived from the primary hepatocytes without any genetic modification (Supplementary information, Figs. S2b–f and 5g).

We found individual variations in the proliferative potential of HepLPCs obtained from different donors. HepLPCs from 7 out of 9 donors could be cultured over 10 passages and 3 of these could be cultured up to 20 passages (Supplementary information, Fig. S3a). Late-passage HepLPCs displayed reduced proliferative capacity, decreased expression of progenitor markers and rare chromosomal abnormalities (Supplementary information, Fig. S3b–d).

### Essential role of SIRT1 in human hepatocyte-to-LPC conversion and expansion

**Fig. 2:**
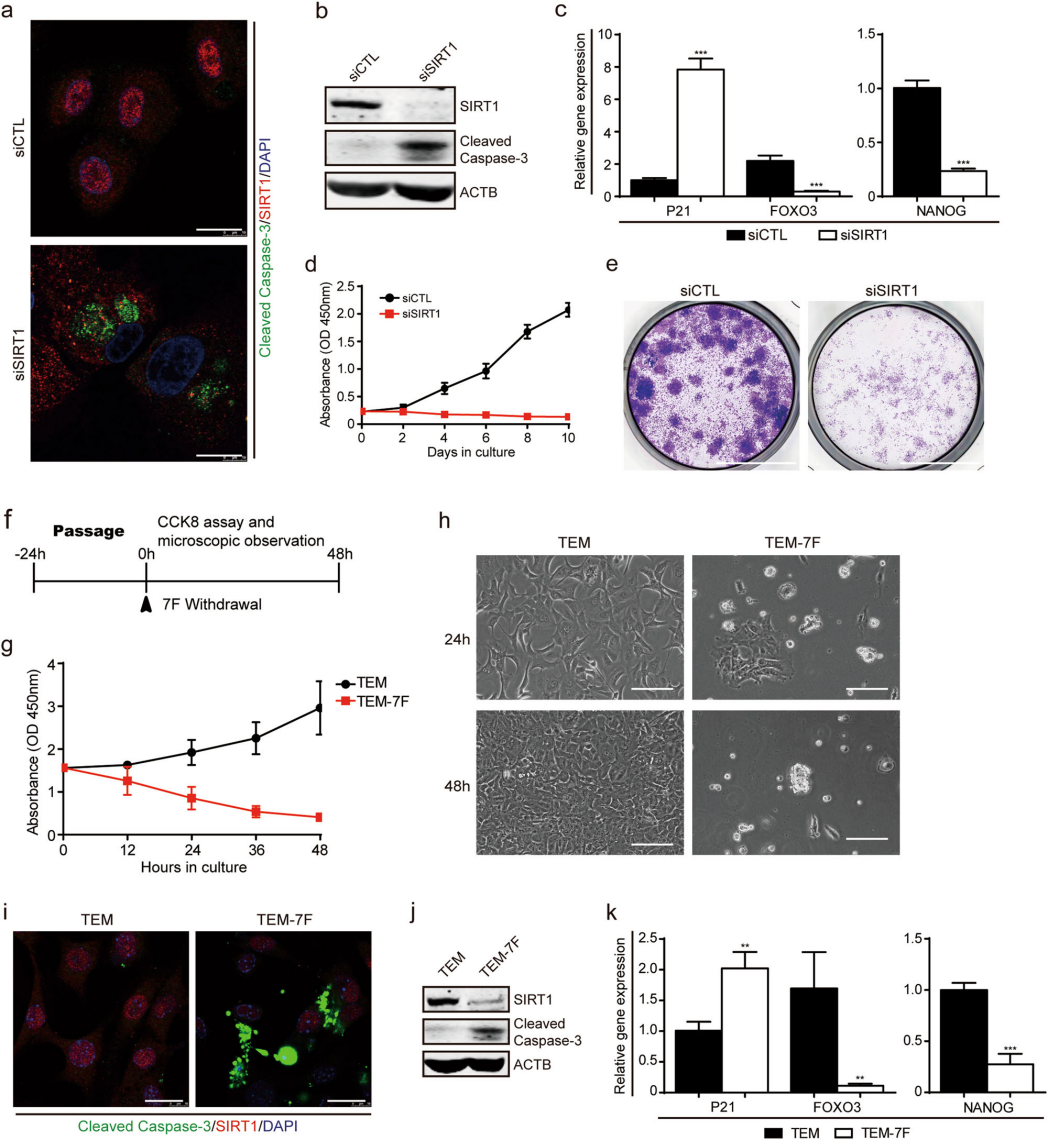
SIRT1 is essential for hepatocyte-to-LPC conversion and expansion. **a** Immunofluorescence images of SIRT1 and cleaved-caspase-3 at day 4 in cells transfected with control siRNA (siCTL) or siRNA-targeting SIRT1 (siSIRT1). Scale bars, 20 μm. **b** Western blot analysis of SIRT1 and cleaved-caspase-3 expression as in (**a**). **c** QPCR analyses for the expression of P21, FOXO3 and NANOG in cells transfected with siCTL or siSIRT1. Error bars represent s.d.; *n* = 3 donors (two-tailed unpaired *t*-test, ***P < 0.001). **d** CCK-8 analyses demonstrating suppression of cell proliferation in the presence of siSIRT1. Error bars represent s.d.; *n* = 5 technical replicates from one donor. **e** Light microscopy images show a reduction in clone formation following inhibition of SIRT1 by crystal violet staining. Scale bars, 10 mm. **f** Schematic of the 7 factors (7 F) withdrawal assay during passage. **g** CCK-8 analyses demonstrating suppression of cell proliferation in the absence of the 7 F within 48 h. Error bars represent s.d.; *n* = 5 technical replicates from one donor. **h** Light microscopy shows reduced cell proliferation in response to the withdrawal of the 7 F at 24 h and 48 h. Scale bars, 100 µm. **i** Immunofluorescence images of SIRT1 and cleaved-caspase-3 in HepLPCs cultured in TEM or TEM-7F at 24 h. Scale bars, 20μm. **j** Western blot analysis of SIRT1 and cleaved caspase-3 expression as in (**i**). **k** QPCR analyses for the expression of P21, FOXO3 and NANOG as in (**i**). Error bars represent s.d.; *n* = 3 donors (two-tailed unpaired *t*-test, **P < 0.01, ***P < 0.001)

Chemically-induced cell reprogramming is usually accompanied by epigenetic changes^10,^ leading us to investigate whether epigenetic modifiers could influence hepatocyte-to-LPC conversion. To our surprise, we found that although none of the commonly used epigenetic modifiers promoted cell growth (Supplementary information, Fig. S4a), hepatocytes incubated with nicotinamide (NAM) displayed dramatically decreased proliferative capacity and gradually underwent cell apoptosis or senescence (Supplementary information, Fig. S4b, c). As NAM is an inhibitor of the sirtunin family, we analyzed gene expression of SIRT1-7 and found that SIRT1 was selectively upregulated under TEM culture conditions (Supplementary information, Fig. S4d). NAM-treated cells showed higher expression of cleaved Caspase-3 and lower expression of SIRT1 than non-treated controls (Supplementary information, Fig. S4e). Furthermore, the expression levels of genes downstream of SIRT1 were altered accordingly, including the cellular senescence gene P21, the oxidative stress gene FOXO3 and the stemness gene NANOG (Supplementary information, Fig. S4f, g). Cells cultured with NAM also showed lower numbers of colonies than those cultured without NAM (Supplementary information, Fig. S4h). These results indicate that NAM inhibits cell proliferation and induces apoptosis possibly through the inactivation of SIRT1. Accordingly, two other SIRT1 inhibitors, Tenovin-6 and EX 527 displayed similar inhibitory effects on cell growth (Supplementary information, Fig. S4i-m). siRNA-mediated SIRT1 silencing also induced drastic cell apoptosis and prevented colony formation (Fig. 2a–e). Withdrawal of seven TEM factors led to the loss of proliferative capacity and induction of apoptotic cell death. It is of note that SIRT1 expression was lost abruptly, further supporting the possibility of a contributory role for SIRT1 in maintaining hepatocyte-to-LPC conversion and expansion (Fig. 2f–k).

### Consistent expression of progenitor-associated genes in HepLPCs

**Fig. 3:**
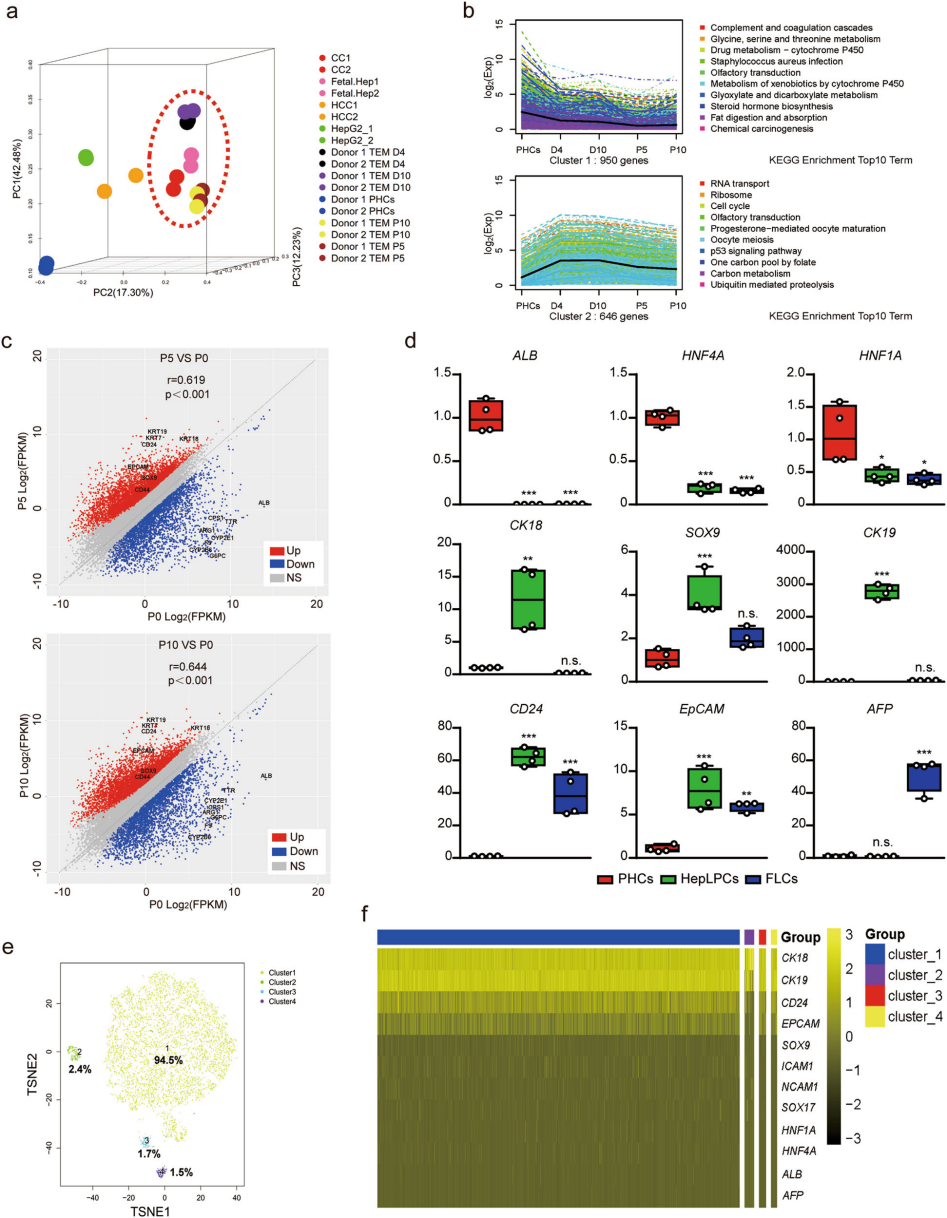
Characterization of HepLPCs. **a** Principal component analysis of PHCs (freshly isolated), hepatic hepatoma cell lines (HepG2), hepatocellular carcinoma (HCC), cholangiocarcinoma (CC), fetal hepatocytes (Fetal.Hep) and HepLPCs at day 4, day 10, passage 5 and passage 10 in TEM from two donors based on global gene expression profile. **b** K-mean analysis of hepatic function related genes (top) and cell cycle related genes (bottom) in PHCs and cells at day 4, day 10, passage 5 and passage 10 in TEM. **c** Correlation scatter plot show the changes of liver progenitor related genes by KEGG enriched analysis. Passage 5 or passage 10 versus PHCs (P0). **d** QPCR analyses for the expression of liver progenitor related genes, PHCs were freshly isolated (one-way ANOVA with Dunnett correction for multiple comparisons, *n* = 4 donors, n.s., non-significant, *P < 0.05, **P < 0.01, ***P < 0.001). **e** *t*-SNE projection of all 7459 individual HepLPCs based on K-means clustering, different colors represent different subgroups. **f** Heatmap of liver progenitor- and hepatic lineage-related genes in different subgroups

To characterise HepLPCs further, we compared global gene expression profiles of HepLPCs from two donors with primary hepatocytes (PHCs), hepatic hepatoma cell lines (HepG2), hepatocellular carcinoma (HCC), cholangiocarcinoma (CC) and fetal hepatocytes (Fetal.Hep, at 19 gestational weeks^11^). Principal component analysis and hierarchical clustering showed that HepLPCs differed from hepatic hepatoma cell lines as much as they did from hepatocellular carcinoma. However, fetal hepatocytes and cholangiocarcinoma clustered together and were clearly separated from hepatic hepatoma cell lines and hepatocellular carcinoma. We note that following hepatocyte-to-progenitor cell conversion, HepLPCs displayed close clustering with fetal hepatocytes in their gene expression profiles (Fig. 3a, Supplementary information, Fig. S5a). A K-mean analysis revealed a significant enrichment in HepLPC gene expression for functions that are associated with RNA transcription, DNA packaging, cell cycle and DNA repair. On the other hand, a set of liver function pathways for the metabolism of xenobiotic substances, lipids and amino acids were significantly reduced during hepatocyte-to-LPC conversion (Fig. 3b). Expression of hepatic nuclear receptors and transcription factors changed accordingly with time (Supplementary information, Fig. S5b, c). Gene set enrichment analysis (GSEA) confirmed a consistent enrichment of genes in proliferating cells not only at passage 5 but also at passage 10 (Supplementary information, Fig. S5d). A correlation scatter analysis indicated that HepLPCs at both early and late passages displayed similar changes in their gene expression profile compared to PHCs (Fig. 3c). Specifically, HepLPCs from 3 donors showed consistent expression of the hepatic marker ALB (14.9% ± 4.0%) and the progenitor marker CK19 (99.6% ± 0.2%) in HepLPCs at both early and late passages (two-tailed unpaired *t*-test, *p* *=* *0.9528* of ALB, *p* *=* *0.4353* of CK19, Supplementary information, Fig. S5e). Furthermore, HepLPCs not only retained the expression of hepatocyte-lineage marker genes including HNF1A, HNF4A, ALB (weakly expressed) and CK18 but also displayed expression of liver progenitor cell markers such as SOX9, CK19, CD24 and EpCAM (Fig. 3d, Supplementary information, Fig. S5f, g). K-means clustering of single-cell RNA sequencing data partitioned HepLPCs into 4 subgroups with overlapping gene expression profiles but differing in network composition and signal strength (Fig. 3e, Supplementary information, Fig. S6a, b). The expression of hepatic lineage markers such as HNF1A, HNF4A, ALB and CK18 were scattered or widely distributed in HepLPCs whereas the progenitor markers (EpCAM, CD24, CK19 and SOX9) were generally expressed at varying levels, suggesting that proliferative HepLPCs acquired liver progenitor features at the transcriptional level (Fig. 3f).

### Efficient hepatic differentiation of HepLPCs

**Fig. 4:**
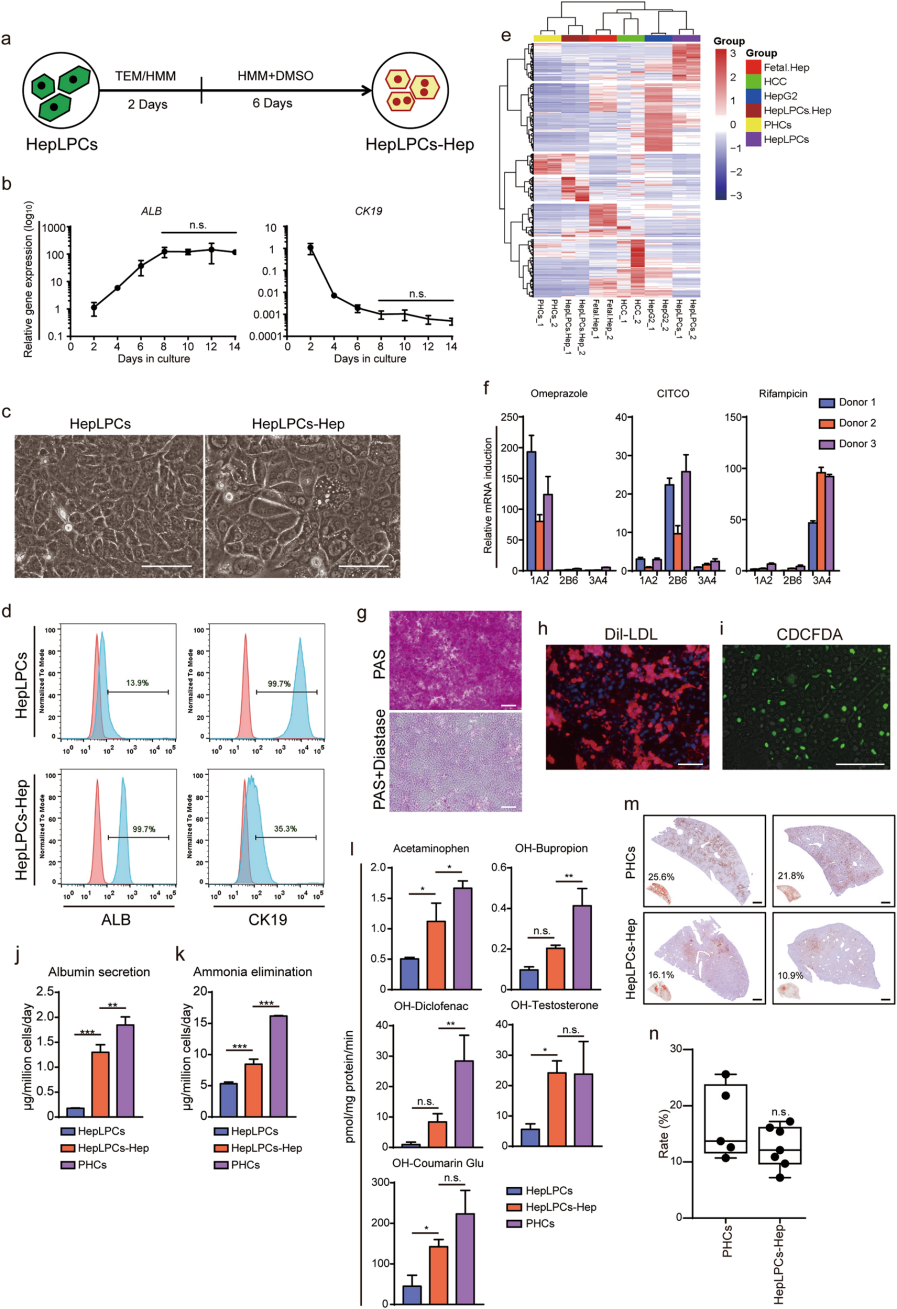
Efficient hepatic differentiation of HepLPCs in vitro. **a** Schematic of the hepatic-differentiation protocol. TEM/HMM, mixed by 1:1. **b** QPCR analyses for the expression ALB and CK19 during hepatic-differentiation of HepLPCs (passage 5) from day 2 to day 14. Error bars represent s.d.; *n* = 3 donors (one-way ANOVA with Tukey correction for multiple comparisons, n.s., non-significant). **c** Light microscopy images of HepLPCs versus HepLPCs-Hep. Scale bars, 50 μm. **d** Flow cytometric analysis showing the proportion of ALB and CK19 positive-cells in HepLPCs or HepLPCs-Hep from donor 4 passage 5. Blue, positive-cells; red, negative controls. (**e**) Euclidean hierarchical clustering of HepG2, HCC, HepLPCs, HepLPCs-Hep and PHCs using differentially expressed genes ( ≥2-fold changes and *P* < 0.001) in HepLPCs versus HepLPCs-Hep. **f** Induction of CYP450 expression in HepLPCs-Hep from three donors (passage 5) in response to stimulation with omeprazole, CITCO, and rifampicin for 72 h. 1A2, CYP1A2; 2B6, CYP2B6; 3A4, CYP3A4. Expression normalized to DMSO-treated controls. Error bars represent s.d.; *n* = 3 technical replicates. **g** PAS staining with or without diastase, **h** DiI-LDL uptake and **i** CDCFDA staining of HepLPCs-Hep. Scale bars, 100 µm. **j** Albumin production and **k** ammonia elimination during 24 h measured in supernatant, PHCs were freshly isolated. Error bars represent s.d.; *n* = 3 donors (one-way ANOVA with Tukey correction for multiple comparisons, **P < 0.01, ***P < 0.001). **l** CYP metabolic activities of HepLPCs, HepLPCs-Hep (passage 5) and PHCs (freshly isolated). The metabolic products of Acetaminophen, OH-Bupropion, OH-Diclofenac, OH-Testosterone and OH-Coumarin Glu determined by liquid chromatography-tandem mass spectrometry according to standard curves. Error bars represent s.d.; *n* = 3 donors (one-way ANOVA with Tukey correction for multiple comparisons, n.s., non-significant, *P < 0.05, **P < 0.01). **m** Representative immunohistochemical staining of hALB of human chimeric mouse liver tissues, PHCs were freshly isolated. Scale bars, 400 µm. **n** Quantification of the repopulation efficiency estimated by hALB-positive foci. No significant difference is noticed between PHCs and HepLPCs-Hep (passage 5). Error bars represent s.d.; n.s., non-significant

Next, we evaluated the hepatic differentiation ability of HepLPCs using our previously reported hepatic maturation medium at day 8^8^ (HMM, Fig. 4a). Compared to undifferentiated cells, hepatocyte-like cells derived from HepLPCs at passage 5 or 10 exhibited similar polygonal morphology with increased ratios of cytoplasm:nucleus and had elevated levels of ALB (increased from 13.9% to 99.7%) and reduced levels of CK19 (decreased from 99.7% to 35.3%) (Fig. 4b–d, Supplementary information, Fig. S7a). Both early- and late-passage HepLPCs retained similar hepatic differentiation capacity (Supplementary information, Fig. S3e). RNA sequencing revealed that the expression profiles of HepLPC-derived hepatocytes clustered with those of primary hepatocytes, especially in hepatic function-related genes (Fig. 4e, Supplementary information, Fig. S8a, b). Accordingly, gene sets related to hepatic functions, including ABC transporters, bile secretion and cytochrome P450 genes were rapidly induced after 8-day upon differentiation (Supplementary information, Fig. S8a, b). Quantitative PCR analyses of nuclear receptors, P450 enzymes and drug transporters validated the findings from RNA-sequencing (Supplementary information, Fig. S7b). Re-differentiated hepatocytes also displayed homogenous expression of hepatic markers, including E-cadherin, CYP3A4, HNF1A, and ALB (Supplementary information, Fig. S7c). Omeprazole stimulation induced CYP1A2 expression by 80 ± 11 to 193 ± 27-fold; CITCO induced CYP2B6 expression by 10 ± 2 to 26 ± 4-fold; and rifampicin treatment induced expression of CYP3A4 by 47 ± 2 to 96 ± 5-fold (over DMSO-treated control, Fig. 4f). Periodic acid Schiff (PAS) reaction, CDCFDA staining and low-density lipoprotein (DiI-LDL) uptake assays showed that HepLPC-derived hepatocytes could accumulate glycogen, form bile canaliculi and take up LDL (Fig. 4g–i). They also produced much higher level of albumin and eliminated ammonia more efficiently than undifferentiated HepLPCs (Fig. 4j, k). Furthermore, the re-differentiated cells were able to metabolize Acetaminophen, OH-Bupropion, OH-Diclofenac, OH-Testosterone and OH-Coumarin Glu (Fig. 4l). To test the ability of HepLPCs (passage 5) to engraft and differentiate into mature hepatocytes in vivo, we transplanted HepLPCs-Hep into Fah^−/−^Rag2^−/−^(F/R) mice with intermittent NTBC treatment.^12^ Immunohistochemical staining using human-specific antibodies showed ALB^+^ human cells as hepatocyte foci covering 7.2%-16.1% of the liver parenchyma in the surviving mice (Fig. 4m, n). Together with the observed increases of serum ALB and AAT, these results suggest further maturation competence of HepLPCs in a three-dimensional (3D) matrix microenvironment (Supplementary information, Fig. S7d, e). To investigate the impact of HepLPCs-Hep on survival, we transplanted the differentiated HepLPCs (passage 5) into F/R mice that we maintained for 2 months without NTBC treatment. We found that HepLPCs-Hep transplantation significantly improved the liver functions of F/R mice and their survival (Supplementary information, Fig. S9a–c).

### Hepatitis B virus infection and reactivation in differentiated 3D spheroids of HepLPCs

**Fig. 5:**
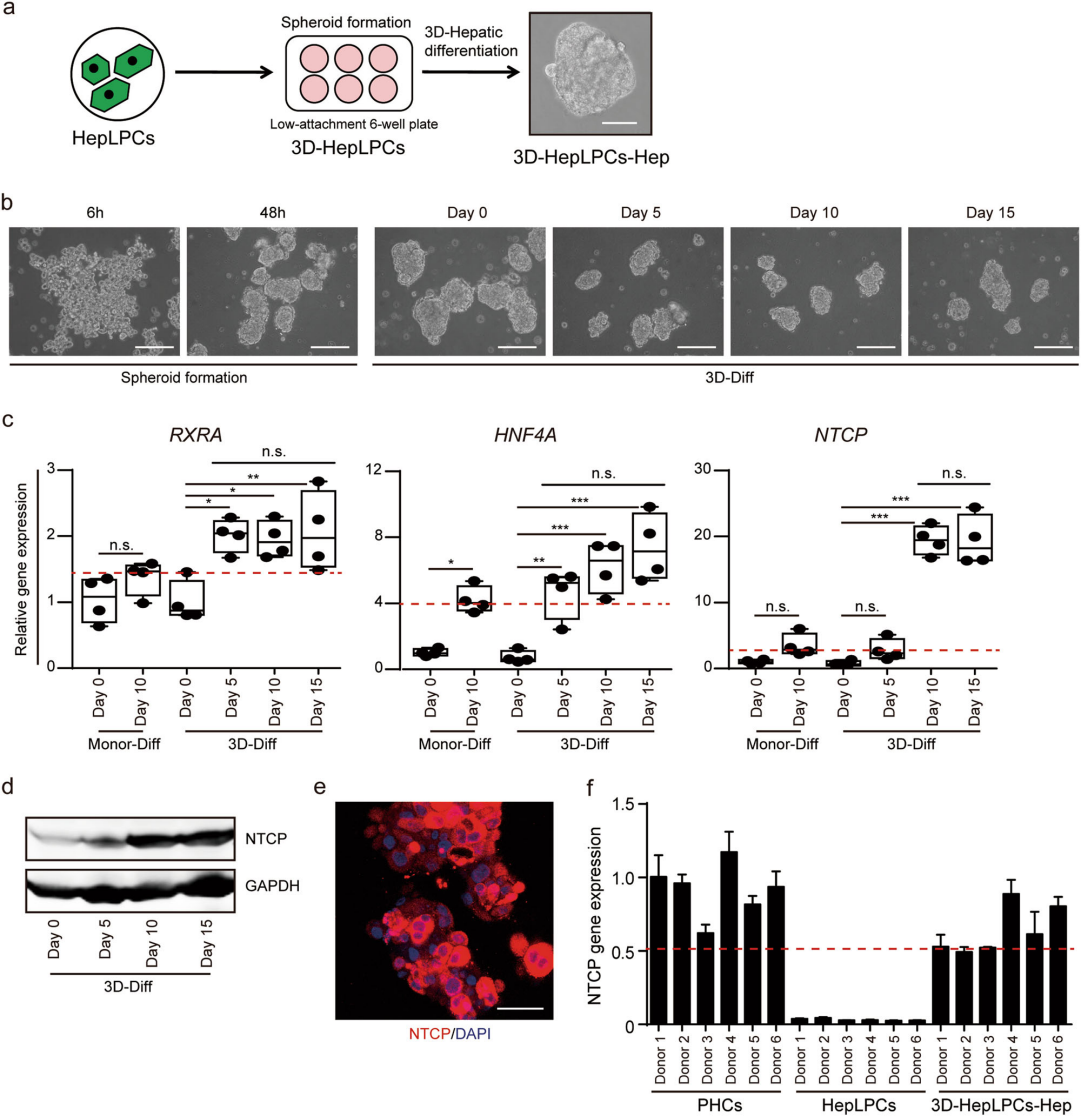
Expression of proviral host factors in 3D-HepLPCs-Hep. **a** Overview of the protocol for formation and differentiation of 3D-HepLPCs. Scale bar, 50 μm. **b** Time course of 3D spheroid formation and hepatic differentiation. 3D-Diff, 3D spheroid differentiation. Scale bars, 200 µm. **c** QPCR analyses for the expression of RXRA, HNF4A and NTCP in HepLPCs (passage 5) with monolayer or 3D differentiation. (one-way ANOVA with Tukey correction for multiple comparisons, *n* = 4 donors, n.s., non-significant, *P < 0.05, **P < 0.01, ***P < 0.001). **d** Western blot analysis of NTCP expression in 3D-HepLPCs with differentiation from day 0 to day 15. **e** Immunofluorescence images of NTCP in HepLPCs with 3D spheroid differentiation. Scale bar, 25 μm. **f** QPCR analyses for the expression of NTCP in PHCs (collected overnight after seeding as a monolayer), HepLPCs and 3D-HepLPCs-Hep from six donors passage 5. Error bars represent s.d.; *n* = 3 technical replicates

Previous studies have shown that 3D culture can enhance the degree of hepatic function similar to events in vivo.^13^ We generated 3D cell spheroids (3D-HepLPCs) from the HepLPCs, and further differentiated these spheroids (3D-HepLPCs-Hep) as a model for HBV infection (Fig. 5a). The cells of a single cell suspension of HepLPCs in low-attachment 6-well plate began aggregating within 6 h and formed cellular spheres after 48 h in TEM/HMM and further differentiation enabled by culture in HMM for 5, 10 and 15 days (Fig. 5b). To determine the utility of 3D-HepLPCs-Hep as an infection model for HBV, we evaluated the expression of known host factors essential for HBV infection and replication, including the transcription factors retinoid X receptor A (RXRA), HNF4A, and the viral receptor NTCP (Fig. 5c). Remarkably, the expression level of NTCP was dramatically elevated by over 19.4 ± 2.2-fold in 3D-HepLPCs-Hep after 10 days of differentiation relative to controls (Fig. 5d, e). Further analyses of the expression kinetics of NTCP during the reversible conversion in cells derived from six donors revealed its expression was downregulated in HepLPCs and could be reinduced upon 3D spheroid differentiation, reaching up to 50% of the level in parental hepatocytes (Fig. 5f).

**Fig. 6:**
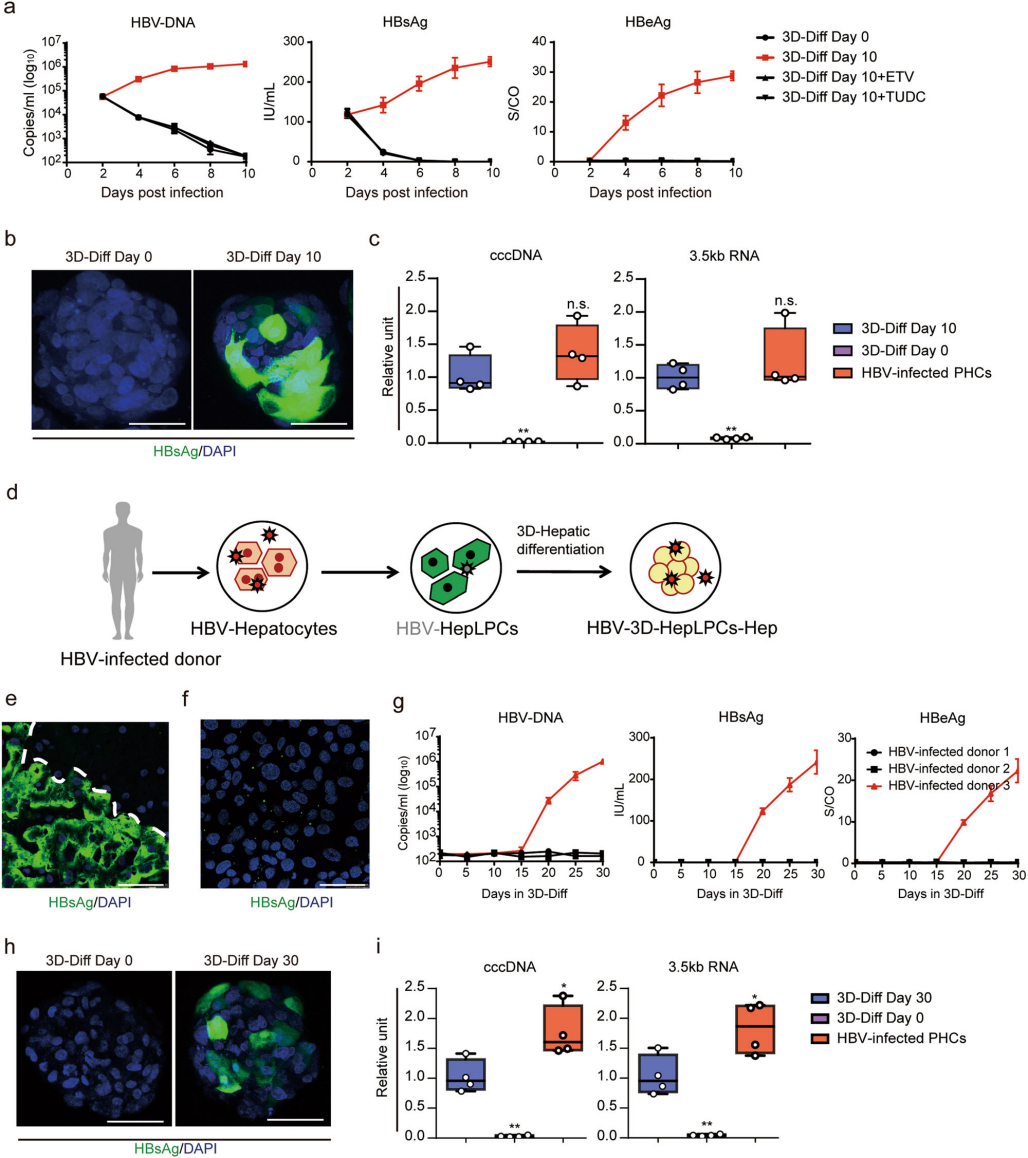
HBV infection and reactivation in 3D-HepLPCs-Hep. **a** Extracellular HBV-DNA and secreted viral antigens were monitored from 2 to 10 days post infection in 3D-HepLPCs treated with ETV or TUDC. Error bars represent s.d.; *n* = 4 donors. **b** HBsAg staining in 3D-HepLPCs with or without differentiation at 8 days post infection. Scale bars, 25 μm. **c** QPCR analyses for the expression of cccDNA and 3.5 kb RNA in 3D-HepLPCs with or without differentiation versus HBV-infected PHCs (10 days post infection*, n* = 4 donors, one-way ANOVA with Dunnett correction for multiple comparisons, n.s., non-significant, **P < 0.01). **d** Schematic of HBV reactivation in 3D-HepLPCs-Hep derived from HBV-infected donor. **e** HBsAg staining of the liver tissue collected from HBV-infected donor and **f** disappearance after 10-day culture in TEM. Scale bars, 50 μm. **g** Extracellular HBV-DNA and secreted viral antigens are monitored from day 0 to day 30 in HMM + DMSO in 3D-HepLPCs derived from three HBV-infected donors, *n* = 3 technical replicates. **h** HBsAg staining of patient-derived 3D-HepLPCs with or without 30-day differentiation. Scale bars, 25 μm. **i** QPCR analyses for the expression of cccDNA and 3.5 kb RNA in patient-derived 3D-HepLPCs with or without 30-day differentiation versus HBV-infected PHCs (10 days post infection, *n* = 4 technical replicates, one-way ANOVA with Dunnett correction for multiple comparisons, *P < 0.05, **P < 0.01)

Given the importance of the spheroid format for maintaining expression of the HBV receptor as well as other hepatocyte functions, we next investigated whether 3D-HepLPCs-Hep support HBV infection. We infected differentiated spheres at days 0 and 10 with HBV-containing sera at a multiplicity of infection (MOI) of 300 in the absence or presence of entecavir (ETV), a reverse-transcription inhibitor or tauroursodeoxycholic acid (TUDC),^14^ a substrate of NTCP that inhibits HBV pre-S1 lipopeptide binding. Extracellular HBV DNA representing newly produced virions, as well as secreted HBV surface antigen (HBsAg) and HBV e antigen (HBeAg) peaked at day 8 and thereafter remained stable (Fig. 6a). Treatment with either of the compounds prevented HBV infection of 3D-HepLPCs-Hep. Furthermore, immunostaining showed that approximately 30% of cells were positive for HBsAg (Fig. 6b, Supplementary information, Movies S3 and S4). The production of 3.5 kb mRNA (the main HBV transcript), and viral transcription template cccDNA were also detected upon infection at levels close to those in HBV-infected PHCs (Fig. 6c). Southern blotting detected HBV cccDNA (Supplementary information, Fig. S10a) and validated the production of infectious HBV virions (Supplementary information, Fig. S10b). These results suggest that the expression kinetics of NTCP were concomitant with HBV infectivity during the reversible conversion between hepatocytes and LPC-like cells.

As human hepatocyte division triggered substantial loss of cccDNA in vivo,^15,16^ we investigated whether hepatocyte-to-LPC conversion and expansion could lead to HBV reduction in vitro (Fig. 6d). Primary hepatocytes were isolated from patients with HBV infection, in which HBsAg was regionally distributed across the liver (Fig. 6e). Accordingly, several such hepatocytes were positive for NTCP but negative for HBsAg (Supplementary information, Fig. S10c). However, after culture in TEM for 10 days, HBV-DNA decreased dramatically and almost none of the expanding cells were expressing HBsAg, suggesting the loss of viral load (Fig. 6f; Supplementary information, Fig. S10d). HepLPCs derived from HBV-infected donors (HBV-HepLPCs) possessed the ability to proliferate and differentiate just as those derived from normal donors (Supplementary information, Fig. S10e–h). Notably, when HBV-HepLPCs underwent redifferentiation in the 3D spheroid format, one of three samples displayed HBV infection markers again at day 15 and these increased throughout a 30-day period (Fig. 6g). The production of HBsAg, cccDNA and 3.5kd RNA could also be detected following 30 days of differentiation (Fig. 6h, i). These results suggest a rebound of HBV replication in HepLPCs upon in vitro 3D differentiation, possibly caused by the persisting cccDNA in these cells.

### Evaluation of 3D spheroid model as a platform for anti-HBV studies

**Fig. 7:**
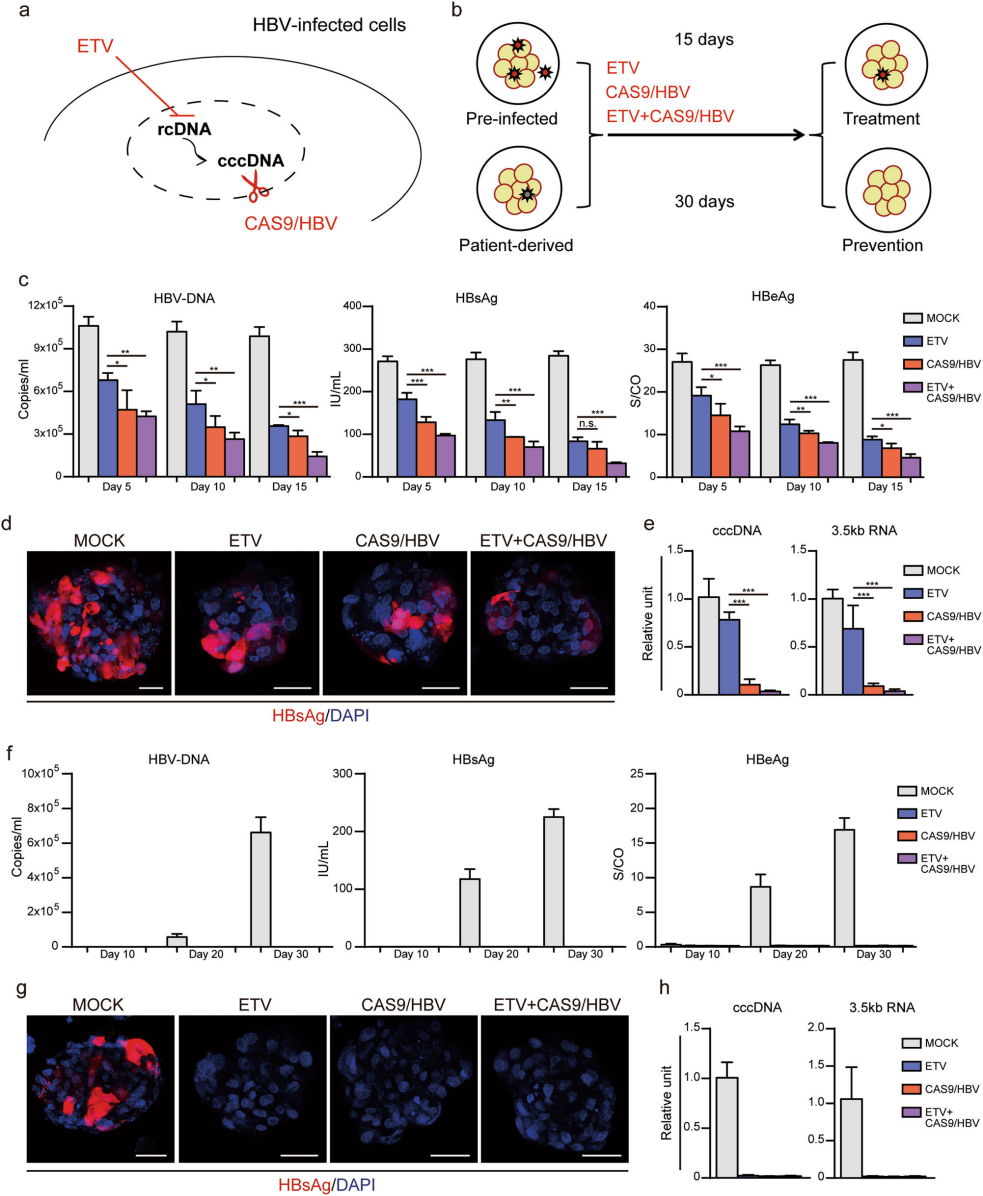
Anti-HBV studies in 3D spheroid models. **a** Schematic of HBV life cycle indicating the presumed mechanism of action of ETV and CAS9/HBV. **b** The antiviral drugs used in pre-infected or patient-derived 3D spheroid models. **c**–**e** The makers’ expression of HBV in pre-infected 3D spheroid models. **c** Extracellular HBV-DNA and secreted viral antigens were monitored after drug treatment in pre-infected 3D spheroid models at different time points. Error bars represent s.d. (one-way ANOVA with Dunnett correction for multiple comparisons*, n* = 4 technical replicates, n.s., non-significant, *P < 0.05, **P < 0.01, ***P < 0.001). **d** HBsAg staining of pre-infected 3D spheroid models treated with the drugs for 15 days. Scale bars, 25 μm. **e** QPCR analyses for the expression of cccDNA and 3.5 kb RNA in pre-infected 3D spheroid models treated with the drugs. Error bars represent s.d. (one-way ANOVA with Dunnett correction for multiple comparisons*, n* = 4 technical replicates, *** P < 0.001). **f**–**h** The makers’ expression of HBV in patient-derived 3D spheroid models. **f** Extracellular HBV-DNA and secreted viral antigens were monitored after drug treatment in patient-derived 3D spheroid models at day 10, day 20 and day 30. Error bars represent s.d.; *n* = 4 technical replicates. **g** HBsAg staining of patient-derived 3D spheroid models treated with the drugs for 30 days. Scale bars, 25 μm. **h** QPCR analyses for the expression of cccDNA and 3.5 kb RNA in patient-derived 3D spheroid models treated with the drugs. Error bars represent s.d.; *n* = 4 technical replicates

In the light of the urgent need for development of antiviral agents, we tested the antiviral effect of ETV and an adenoviral vector expressing the validated Cas9/HBV-sgRNAs^17^ (CAS9/HBV, Supplementary information, Fig. S11a–c), either independently or in combination, in our pre-infected or patient-derived 3D spheroid models (Fig. 7a, b). We first explored their efficacy in the pre-infected 3D spheroid model. Although both ETV and CAS9/HBV induced suppression of HBV DNA release, HBsAg and HBeAg secretion and expression, they showed enhanced antiviral effects in combination (Fig. 7c, d). More importantly, CAS9/HBV alone or in combination with ETV robustly prevented production of cccDNA and 3.5 kb mRNA whereas monotherapy with ETV only partially reduced their levels (Fig. 7e). Next, we evaluated their therapeutic effects in preventing HBV reactivation in the patient-derived 3D spheroid model. The sample with HBV reactivation potential was cultured in suspension to generate 3D spheroids. ETV, CAS9/HBV or their combination was administered at an early stage of spheroid formation before HBV reactivation. Remarkably, all treatments completely prevented the rebound of HBV replication (Fig. 7f–h).

## Discussion

The reversible hepatocyte-to-ductal metaplasia observed in vivo has provoked us and others to generate culturable progenitor cells from mouse hepatocytes with defined chemical factors.^7,8^ Here, we further show that human hepatocytes can be efficiently converted to progenitor-like cells in culture. Technically, about 0.1–1.5 billion HepLPCs can be generated in 30 days from 20 thousand PHCs of different donors. These cells differentiate into functional hepatocytes in vitro and engraft into the liver parenchyma upon transplantation. Extensive analysis of genome-wide expression profiles demonstrated that the expanded cells clustered closely with human fetal liver cells, suggesting that the cultured mature hepatocytes undergo dedifferentiation towards a fetal state.

Conversion of human hepatocytes to progenitor-like cells *in vitro* is a highly efficient process, in which hepatocytes can acquire LPC characteristics in 3-4 days and then proliferate exponentially for weeks. The conversion is a process of epigenetic remodeling that not only shapes cell identity but also controls the cellular response to intrinsic and extrinsic stresses.^18,19^ We show that the inhibition of NAD^+^-dependent deacetylase SIRT1 completely prevented human hepatocyte-to-LPC conversion and led to apoptotic cell death, suggesting that SIRT1-mediated epigenetic modification at the targeted endogenous loci is essential for proper cell reprogramming. In support of this notion, SIRT1 has been shown to be required for maintenance of telomere integrity and genomic stability during iPS cell induction.^20^ Furthermore, we found that NANOG expression can be regulated by SIRT1 in hepatocyte-derived progenitors, as was also noticed in embryonic stem cells.^21^ Together, these lines of evidence indicate that SIRT1 plays important roles in the maintenance of stemness mediated through pluripotency factors. It is of note that the SIRT1 inhibitor seems not to prevent conversion of hepatocytes to progenitor-like cells in mice. However, the methodology failed to reprogram human hepatocytes.^7^ These results suggest a species-specific role of SIRT1 during hepatocyte-to-LPC conversion. The mechanisms underlying the discrepancies await further clarification.

Consistent with the properties of their mouse counterparts, human HepLPCs could efficiently convert back to the mature hepatic state upon in vitro differentiation. More importantly, when cultured in suspension with gentle rotation, they preferably form spheroids and display enhanced liver-specific functions. In line with these findings, the pronounced induction of NTCP expression as well as other factors responsible for HBV infection and replication, such as RXRA and HNF4A,^22^ was observed in 3D spheroid cultures. It has been known that the lack of a robust cell culture system permissive for hepatitis virus infection has limited virus research and drug discovery. By using hepatosphere cultures, we provide a novel in vitro model for HBV infection which fully support the major steps of viral life cycle. A recent report showed that proliferation of primary human hepatocytes could deplete nuclear cccDNA in vivo.^16^ Consistent with this observation, we showed that the HBV-infected human hepatocytes under went a substantial loss of HBV cccDNA during hepatocyte-to-LPC conversion and expansion in vitro, and this was accompanied by reduced expression of genes required for HBV entry and replication.^9,23^ Remarkably, the silenced HBV could be reactivated upon hepatosphere differentiation, indicating that a fraction of the cccDNA was refractory to further clearance. These findings support the in vivo evidence that a reservoir for HBV reinfection lay within a few persistently infected cells.^16^ Further characterization of these cells in vitro and in vivo may promote development of therapeutic strategies to achieve viral elimination.

Since patient-specific HepLPCs can be readily generated from the minimally invasive liver biopsy samples, this model is highly suitable for studying donor-to-donor variations in virus-host interaction including patients with genetic polymorphisms or carrying HBV with mutations.^24^ It may also be used for screening novel antiviral agents and testing antiviral drugs in the individualized HBV treatment. Although the single use of an antiviral agent failed to deplete HBV cccDNA in 3D-cultured spheroids, its combination with CRISPR/Cas9-based gene editing targeting cccDNA eliminated the vast majority of episomal DNA and prevented viral reactivation, which may represent a significant step towards the cure of chronic HBV infection. In addition, our results show that HepLPCs can be generated highly efficiently without need for introducing exogenous genes and these cells can fully mature and proliferate for months after transplantation. These findings establish such cells as providing a promising, safe pathway towards autologous cell therapy of human liver diseases through transplanting expanded hepatocytes from liver biopsy of individual patients. In conclusion, human HepLPCs offer a highly physiological model system for modelling hepatic disease in vitro and it will be of great interest to determine whether they could become a suitable cell source for autotransplantation in the treatment of end-stage liver diseases.

## Materials and methods

### Human primary hepatocyte isolation and purification

Liver tissues (1–5 g) were obtained from the excised normal tissues adjacent to the hemangioma performed at Eastern Hepatobiliary Surgery Hospital, Shanghai (See Supplementary information, Table S1 for detailed information). The Medical Ethical Council of Eastern Hepatobiliary Surgery Hospital approved the use of this material for research purposes, and informed consent was provided from all patients. Primary human hepatocytes were isolated from these tissues by a modified two-step collagenase perfusion technique that involved enrichment prior to culturing using Percoll isodensity purification.^25^ Briefly, the tissue was pre-perfused with calcium-free buffer (5 mg/ml BSA (Sigma-Aldrich), 0.5 mM EGTA, 1 × Hanks without Ca^2+^ and Mg^2+^, for 15-30 min at 37 °C) and then perfused with collagenase (1 mg/ml collagenase type IV (Sigma-Aldrich), 1 × Hanks with Ca^2+^ and Mg^2+^, for 20-40 min at 37 °C). The digestion was stopped by adding cold DMEM/F12 (Invitrogen) + 1% FCS (Invitrogen) to 30 ml in 50 ml centrifuge tube. The suspension was then filtered through a 100 μm Nylon cell strainer, centrifuged for 3 min at 70 g and resuspended in 35 ml cold DMEM/F12 + 13.5 ml Percoll (GE healthcare, density 1.130 g/ml) + 1.5 ml 10 × HBSS. Cells were pelleted at 100 g for 10 min and washed 3 times in cold DMEM/F12. Further, hepatocytes were purified by fluorescence-activated cell sorting (FACS) (MoFlo XDP, Beckman) to exclude CD24^+^ or EpCAM^+^ progenitor cells. Hepatocytes were incubated with FITC-conjugated anti-human EpCAM antibody (Biolegend) and Alexa Fluor® 488-conjugated anti-human CD24 antibody (Biolegend), synchronously, at 4 °C for 30 min. After staining, cells were sorted by FACS. FITC Mouse IgG2b (Biolegend) and Alexa Fluor® 488 Mouse IgG2a (Biolegend) were used as isotype controls.

### Cell expansion

Viability of purified hepatocytes was around 90% as determined by Trypan blue (Sigma-Aldrich). The cells were plated on a Matrigel-coated (Corning) culture dish at 0.5-2 × 10^4^ cells per cm^2^ and cultured in modified TEM.^8^ This was based on Advanced DMEM/F12 (Invitrogen) supplemented with N2 and B27 (Both from Invitrogen), 1 mM sodium pyruvate (Invitrogen), 10 μg/ml ascorbic acid (Sigma-Aldrich) and the following factors: 20 ng/ml HGF, 20 ng/ml EGF (both from Peprotech), 10 μM Y27632, 3 μM CHIR99021, 1 μM A8301 (all from TargetMol), 1 μM S1P and 5 μM LPA (both from Santa Cruz). 6-12 days after seeding, clonal cells were passaged at a ratio of 1:3-6 after dissociation with Accutase (eBioscience). Medium was changed every other day. To assess population growth and doubling time, 1000 cells were seeded on 6-well Matrigel-coated plates and counted on the designated days, following dissociation with TrypLE Express (Invitrogen) to give single cells. In each experiment, the cell number was determined based on 3 technical replicates. Population doubling time was calculated using the web tool provided by http://www.doubling-time.com/compute.php.

### Cell differentiation

For rapid hepatic-differentiation, HepLPCs were seeded and maintained for 2-4 days in TEM until 90% confluence as described above. Then, half of the medium was changed to Hepatocyte Maturation Medium (HMM).^8^ Cells were kept in TEM/HMM (1:1) for 2 days, and then changed to HMM + 1–2% DMSO (Sigma-Aldrich) for another 6 days for further maturation. HMM was based on advanced DMEM/F12 supplemented with N2 and B27, 10 μM DAPT (TargetMol), 20 ng/ml OSM (Peprotech), 10 μM Dexamethasone (Sigma-Aldrich), 10 μM SB431542 (TargetMol). Medium was changed every day. For 3D spheroid hepatic-differentiation, 1–2 × 10^6^ HepLPCs were seeded in the low-attachment 6-well plate. Cells began aggregating within 6 h and formed cellular spheres after 48 h in TEM/HMM (1:1). The medium was then changed to HMM for 8–10 days to allow further maturation. Frozen stocks were prepared using LiveCyte^TM^ (CryowiseMed, Shanghai). Light microscopy images were captured with an Olympus IX70 camera. These cells were regularly tested negative for mycoplasma contamination.

### Time-lapse imaging and colony formation assay

PHCs were seeded at 20,000 cells/cm^2^ on 6-well Matrigel-coated plates with HGM or TEM. The medium was changed at day 1 and day 2. Time-lapse imaging was then performed using a Lionheart™ FX Fluorescence Microscope (BioTek). Imaging was performed from D2 to D8 at 20 min-intervals, for a total of 500 times for each sample, and a movie was generated for each analyzed field. In order to calculate the conversion efficiency, we counted cell divisions of approximately 50 cells in each movie (*n* = 5) with TEM. For the colony formation assay, 2,000 PHCs were seeded on 6-well Matrigel-coated plates and stained with crystal violet (Sigma-Aldrich) at day 10. Whole-well scanning were performed using the Lionheart™ FX microscope.

### EdU detection

EdU detection was performed using the Cell-Light™ EdU Apollo®488 In Vitro Imaging Kit (RiboBio, Guangzhou). HepLPCs, stained at passage10, were mounted with 1 × Hoechst33342 and photographed with the Olympus IX70.

### Karyotyping analysis

Cultured HepLPCs at different passages in exponential growing phase were incubated with 100 ng/mL colcemid for 40 min at 37 °C. Cultures were then washed and dissociated into single cells using Accutase and processed as described above. Karyotyping was performed at the Karyotype analysis department of Beijing Biocytogen. Chromosomes from at least 40 metaphase-arrested cells were counted.

### Lineage tracing

A lentivirus vector carrying TBG-EGFP-T2A-puro was generated in which GFP and a puromycin resistance gene are driven by a liver-specific promoter and are expressed only in hepatocytes when cultured in hepatocyte maintenance medium (Lonza). Twenty-four hours after infection, puromycin was administered to eliminate uninfected cells and progenitor cells in which TBG promoter is inactive. After 2 days of puromycin treatment, the media were changed to TEM and the expression of GFP was monitored by using fluorescence imaging or FACS analysis.

### Cell counting kit-8 (CCK-8) assay

To evaluate cell proliferation, 1,000 cells were seeded onto 96-well Matrigel-coated plates with conditioned medium for 12 h and then with medium containing 10% (v/v) CCK-8 (Dojindo) for 1 h. Testing was carried out every other day. Proliferation was determined by absorbance measurement at 450 nm using a multimode reader Synergy2 (BioTek).

### SA-β-gal staining

For SA-β-gal staining, PHCs were seeded at 40,000 cells/cm^2^ on 6-well Matrigel-coated plates with HGM or TEM. After 6 days of culture, these cells were fixed in 4% Paraformaldehyde and subjected to SA-β-gal staining using a Cell Senescence SA-β-Gal Staining Kit (Beyotime).

### SIRT1 RNA interference experiments

PHCs were seeded onto 6-well Matrigel-coated plates into TEM. The medium was changed after 12 h. Cells were then transfected with 100 nM SignalSilence® Control siRNA (siCTL, Cell Signaling Technology) or SignalSilence® SIRT1 siRNA (siSIRT1, Cell Signaling Technology) using Lipofectamine 2000 (Invitrogen) according to the manufacturer’s instructions.

### Immunohistochemistry, immunofluorescence and flow cytometry

Cells, 3D spheroid models and tissues were fixed with 4% paraformaldehyde (Sigma-Aldrich) or formalin respectively, and prepared and stained with antibodies, H&E or PAS using routine protocols. The antibodies and dilutions used are listed in Supplementary Information, Table S2. Representative images of H&E and immunohistochemical staining were captured with a Leica Aperio AT Turbo. Images of Immunofluorescence staining were captured with a Leica TCS SP8. PAS images were captured with an Olympus IX70. Single-cell suspensions were fixed with 4% Paraformaldehyde at 4 °C for 10 min and then incubated with the primary antibodies, followed by the secondary antibodies. Flow cytometry analysis was performed using the Beckman MoFlo XDP.

### Quantitative real-time PCR (QPCR)

Total RNA of cells and 3D spheroids were extracted using TRIzol reagent (Invitrogen) according to the manufacturer’s protocols. Real-time PCR analyses were performed using a LightCycler^®^ 96 Real-Time PCR System (Roche) and SYBR Green PCR kit (Roche). Gene transcription was evaluated using the ∆∆Ct method normalized to the housekeeping gene actin beta (ACTB). Primer sequences are respectively listed in supplementary information, Table S3. RNA from fetal human liver was obtained from Department of Cell Biology, Second Military Medical University.

### Western blot

Cells were lysed in RIPA Lysis and Extraction Buffer and protein concentrations were measured using a Pierce™ BCA Protein Assay Kit (Both from Thermo scientific). Proteins were subject to electrophoresis on 8–10% Bis-Tris protein gels and transferred to nitrocellulose membranes (GE healthcare), which were incubated with the primary antibodies, followed by a fluorescently-conjugated secondary antibody. The antibodies and dilutions used are listed in Supplementary Information, Table S2. Finally, the fluorescence density on nitrocellulose membranes was measured on Odyssey CLx Western Blot Detection System (LI-COR Biosciences).

### RNA sequencing and bioinformatic analysis

Total RNA was isolated using RNeasy mini kit (Qiagen, Germany). Pairedend libraries were synthesized by using the TruSeq® RNA Sample Preparation Kit (Illumina, USA) following TruSeq® RNA Sample Preparation Guide. RNA sequencing was performed using the HiSeq system (Illumina) at Shanghai Biotechnology Corporation. Raw sequencing reads were preprocessed by filtering out rRNA reads, sequencing adapters, short-fragment reads and other low-quality reads using Seqtk (https://github.com/lh3/seqtk). We used Hisat2 (version:2.0.4)^26^ to map the cleaned reads to the hg38 reference genome with default parameters. Read counts of each gene were summarized using HTSeq^27^ and normalized using the TMM (trimmed mean of M values) method. edgeR was employed to detect differentially expressed genes (Fold_Change > 2, q.value Benjamini et al.1995 < 0.001). Original data were uploaded to the Gene Expression Omnibus database (accession number GSE105019). The following transcriptomic data were included for gene expression profile analyses: hepatic hepatoma cell lines (HepG2, GSM2205676 and GSM2205677), hepatocellular carcinoma (HCC, GSM2794843 and GSM2794845), cholangiocarcinoma (CC, GSM2653831 and GSM2653832) and fetal hepatocytes (Fetal.Hep, GSM1707674 and GSM1707675).

### Data processing of 10 × genomics single cell gene expression

Mapping to GRCh38 human genome, quality control and read counting of Ensembl genes was performed with cellranger software using default parameters (v2.1.0). Cells with greater than or equal to 200 genes and less than or equal to 10% percentage of mitochondrial genes detected were retained for subsequent analyses. Normalization, dimensionality reduction and clustering of single cells were also performed by cellranger. Using top ten principal components, we reduced the dimensionality by *t*-Distributed Stochastic Neighbor Embedding (tSNE). Cell clustering was performed using the graph-based clustering algorithm which requires building a sparse nearest-neighbor graph (where cells are linked if they fall among the k nearest Euclidean neighbors of one another), followed by Louvain Modularity Optimization (Blondel, Guillaume, Lambiotte, & Lefebvre, 2008). The Seurat-Bimod statistical test was used to identify differentially expressed genes between each group of cells and other groups of cells (FDR < = 0.05 and |log2 Fold Change| > = 2). KEGG pathway enrichment analysis was performed using the Hypergeometric test in R. KEGG pathways were selected by a threshold FDR (adjusted P-value) ≤ 0.05. Original data were uploaded to the Gene Expression Omnibus database (accession number GSE116113).

### Functional hepatocyte studies

For the intake of ac-LDL, cells were incubated with DiI-ac-LDL (Invitrogen) for 6 h, with 1 × Hoechst33342 (Beyotime) for another 30 min, and then captured with the Olympus IX70. To assess secretion of human Albumin, supernatants of HepLPCs-Hep were collected after 24 h of culture. PHCs were seeded on 12-well plates and maintained in HMM for 24 h before collection of culture supernatants. These supernatants were assessed using a Human Albumin ELISA kit (Bethyl Laboratory). To assess ammonia elimination, cells were incubated in HMM supplemented with 3 mM NH_4_Cl. Supernatants were collected 24 h after NH_4_Cl induction. NH4^+^ concentrations were measured using the enzymatic colorimetric assays (Megazyme International) and data were corrected for time and cell numbers. To determine functional polarization, HepLPCs-Hep were incubated for 20 min with 5 μM 5(6)-carboxy-2’, 7’-dichlorofluorescein diacetate (CDCFDA), subsequently washed with ice-cold PBS containing calcium and magnesium and imaging was performed using a Leica TCS SP8.

To characterize CYP450 status, HepLPCs, HepLPCs-Hep and PHCs were cultured in medium containing the substrates: phenacetin/CYP1A2, bupropion/CYP2B6, diclofenac/CYP2C9, and testosterone/CYP3A4, 7-hydroxycoumarin/UGT in 200 μl incubation medium at indicated concentrations for 60 min at 37 °C (conducted in RILD Biotech., Shanghai). To stop the reaction, 800 μl cold methanol was added and the mixture centrifuged. The supernatants were collected for measurement of the indicated productions by LC–MS/MS (Agilent 1200 HLPC and ABI 4000 mass spectrometer). The amount of total cell protein was used to normalize the data.

### Transplantation

Fah^−/−^Rag2^−/−^mice were maintained with drinking water containing NTBC (7.5 μg/ml) which was withdrawn one week before cell transplantation. One day prior to transplantation, mice received 50 mg anti-asialo GM1 (Wako Pure Chemical Industries) per animal via IP injection. 2 × 10^6^ HepLPCs-Hep or PHCs in 150 μl of William E medium were injected into spleens without NTBC administration. Animals received 7.5 μg/ml FK506　(Astellas Ireland) in drinking water and were injected weekly with 50 mg anti-asialo GM1 per animal until the end of the experiment. In in vivo differentiation experiments, mice were intermittently replenished with NTBC depending upon on their body weight. In the survival experiments, mice were withdrawn NTBC treatment and maintained for 2 months without NTBC. After the experiments, the animals were sacrificed and their livers were taken for further analysis. Procedures involving mice were approved by the Institutional Animal Care and Use Committee at the Second Military Medical University.

### HBV infection of 3D-HepLPCs-Hep

Plasma from multiple anonymized patients positive for HBV but negative for HCV and HIV was obtained from Eastern Hepatobiliary Surgery Hospital. Immediately after collection, the virus stock was divided in aliquots and stored at −80 °C until use. For all experiments presented in this study, the same combined isolate of plasma was used. Viral titer was determined by real-time PCR performed on an ABI 7500 (Life Technologies Corporation). For infection, 3D-HepLPCs-Hep were inoculated at a multiplicity of infection (MOI) of 300 in HMM containing 1% DMSO and 4% PEG 8000 (Sigma-Aldrich) for 24 h. At the end of the incubation period, cells were washed three times with HMM. Every 24 h, medium was collected and stored at −80 °C for subsequent analyses and replaced with fresh medium.^22,28^

### Detection of HBsAg and HBeAg

Secreted HBsAg and HBeAg levels in cell supernatants were determined using the respective architect HBsAg Kit (6c36) and the architect HBeAg Reagent kit (6c32) in Architect i2000SR, following the manufacturer’s instructions.

### cccDNA detection

Total DNA was extracted using a QIAamp DNA blood mini kit (Qiagen) according to the manufacturer’s protocol. To assess cccDNA by qPCR, DNA samples were treated with 500 U/ml T5 exonuclease (NEB) at 37 °C for 30 min. Following enzyme inactivation at 70 °C for 30 min, the samples were subjected to QPCR.^29^ For Southern blot analysis, a modified Hirt method was used to extract protein-free vial DNAs as described.^30^ To confirm the nature of cccDNA, the samples were heat denatured at 95 °C for 10 min with or without subsequent EcoRI digestion to linearize the DNA. Agarose gel electrophoresis and Southern blotting were performed as previously described.^31^

### HBV drug treatment experiments

Pre-infected 3D spheroid models were developed as described above (8 days post infection) and then treated with ETV, CAS9/HBV (Adenoviral expression of Cas9/sgRNAs targeting HBV cccDNA) or ETV + CAS9/HBV for 15 days. The two validated sgRNAs were from the reported literature^17^ and inserted into the vector pAdeno-U6-spgRNA v2.0-CMV-3Flag-spCas9/HBV as the adenovirus shuttle vector. The recombinant virus was packaged with the adenovirus backbone plasmid pPE3-GFP by Obio Technology, Shanghai. Adenovirus was introduced into the pre-infected 3D spheroid models for 8 h at a MOI of 60 and entecavior (0.5 μM, TargetMol) was present throughout the 15-day treatment. For the HBV reactivation experiment, patient-derived 3D spheroid models were treated with ETV, CAS9/HBV or ETV + CAS9/HBV to determine their preventive effects on HBV resurgence. During the treatment, adenovirus was added at day 0 and day 15 at a MOI of 60. Entecavior (0.5 μM, TargetMol) was present throughout the 30-day treatment.

### MOI determination

To determine cell numbers in spheroids, we seeded the same number of cells (1.5 × 10^6^) into two parallel wells. Once 3D-HepLPCs had formed, spheroids in one well were collected and incubated with TrypLE Express (GIBCO) until the spheroid had dispersed into single cells and the cells counted. The MOI was then determined by dividing the number of virions added by the cell number.

### Accession numbers

The accession number for the gene arrays reported in this paper is GEO: GSE105019 and GSE116113.

### Statistical analysis

All statistical analyses were performed using GraphPad Prism 7. For comparison between two mean values, a two-tailed unpaired *t*-test was used to calculate statistical significance. For comparison between multiple values, one-way ANOVA was used with Dunnett correction for multiple comparisons when comparing multiple values to a single value or Tukey correction for multiple comparisons when comparing multiple values to each other. A p-value less than 0.05 was considered statistically significant.

## Electronic supplementary material

Supplementary information, Figure S1

Supplementary information, Figure S2

Supplementary information, Figure S3

Supplementary information, Figure S4

Supplementary information, Figure S5

Supplementary information, Figure S6

Supplementary information, Figure S7

Supplementary information, Figure S8

Supplementary information, Figure S9

Supplementary information, Figure S10

Supplementary information, Figure S11

Supplementary information, Table S1

Supplementary information, Table S2

Supplementary information, Table S3

Supplementary information, Movie S1

Supplementary information, Movie S2

Supplementary information, Movie S3

Supplementary information, Movie S4

Supplementary movie legend
