# Supplementary material for: Expansion and differentiation of human hepatocyte-derived liver progenitor-like cells and their use for the study of hepatotropic pathogens
Source: Cell Res. 2018 Oct 25;29(1):8–22. doi: 10.1038/s41422-018-0103-x (PMC6318298; doi:10.1038/s41422-018-0103-x)
Supplement: Supplementary file 1 — Supplementary information, Figure S1 [file 41422_2018_103_MOESM1_ESM.pdf]

Fig. S1

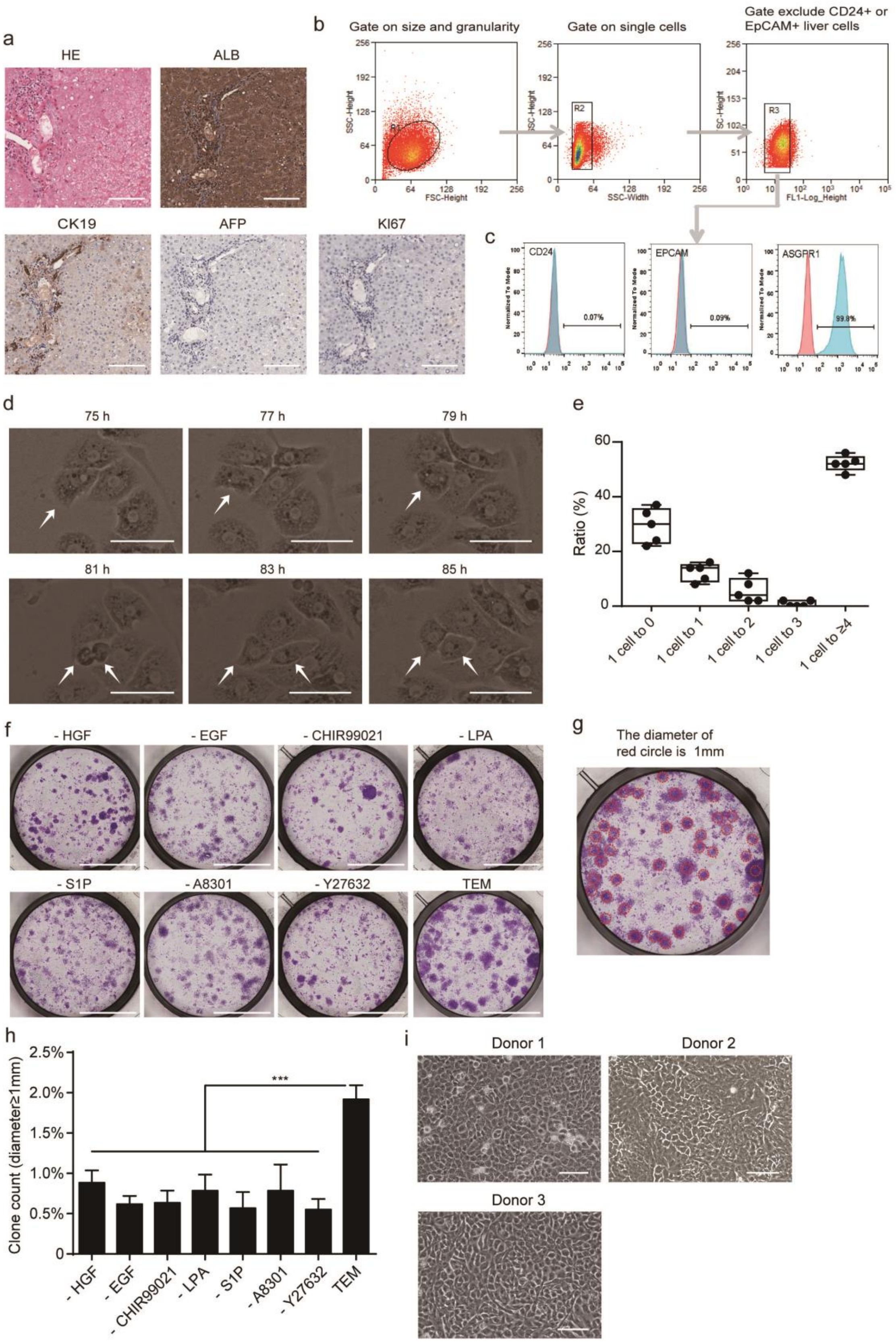
a
HE
ALB
CK19
AFP
KI67
b
Gate on size and granularity
Gate on single cells
Gate exclude CD24+ or EpCAM+ liver cells
SSC-Height
FSC-Height
SSC-Width
FL1-Log_Height
R1
R2
R3
c
CD24
0.07%
EPCAM
0.09%
ASGPR1
99.8%
Normalized To Mode
d
75 h
77 h
79 h
81 h
83 h
85 h
e
Ratio (%)
1 cell to 0
1 cell to 1
1 cell to 2
1 cell to 3
1 cell to ≥4
f
- HGF
- EGF
- CHIR99021
- LPA
- S1P
- A8301
- Y27632
TEM
g
The diameter of red circle is 1mm
h
Clone count (diameter≥1mm)
***
- HGF
- EGF
- CHIR99021
- LPA
- S1P
- A8301
- Y27632
TEM
i
Donor 1
Donor 2
Donor 3

Supplementary information, fig. S1 Isolation and culture of PHCs in TEM, related to Fig. 1. (a) H&E staining and immunohistochemical staining of ALB, CK19, AFP and Ki67 in serial sections of the donated liver tissues. Scale bars, 100μm. (b) Sorting strategy to purify hepatocytes to exclude CD24 or EpCAM positive progenitor cells using flow cytometry. (c) Flow cytometric analysis showing the proportion of CD24, EpCAM and ASGPR1 positive cells in the populations. Blue, positive cells; red, negative controls. (d) Time-lapse imaging for hepatocytes division in TEM from 75 h to 85 h. White arrows indicate a single cell with two nuclei dividing into two daughter cells. Scale bars, 50μm. (e) The frequency of single cells that produced daughter cells at the end of time-lapse imaging at day 8. Approximately 50 cells observed in each independent experiment, n = 5 donors. (f) Crystal violet staining of clones in TEM or TEM without HGF, EGF, CHIR99021, LPA, S1P, A83-01 or Y27632, respectively. Scale bars, 10mm. (g) Strategy of clone counts. Those with clone diameter more than 1mm are counted. (h) Percentage of colony formation efficiency in TEM or TEM without factors. Error bars represent s.d.; n = 3 donors (one-way ANOVA with Dunnett correction for multiple comparisons, *** $p<0.001$). (i) Light microscopy images of HepLPCs derived from 3 donors in TEM at passage 10. Scale bars, 100μm.
