# Supplementary material for: Expansion and differentiation of human hepatocyte-derived liver progenitor-like cells and their use for the study of hepatotropic pathogens
Source: Cell Res. 2018 Oct 25;29(1):8–22. doi: 10.1038/s41422-018-0103-x (PMC6318298; doi:10.1038/s41422-018-0103-x)
Supplement: Supplementary file 2 — Supplementary information, Figure S2 [file 41422_2018_103_MOESM2_ESM.pdf]

Fig. S2

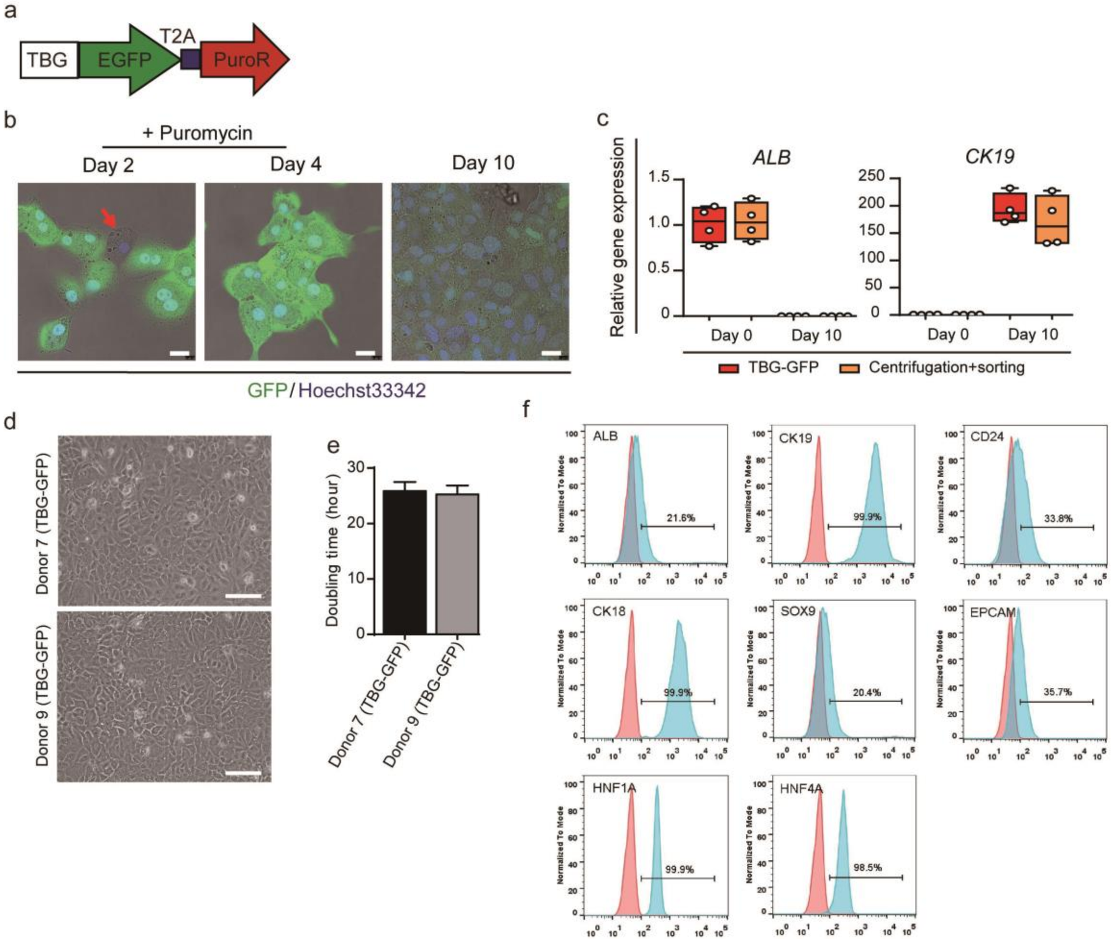
a
TBG
EGFP
T2A
PuroR
b
+ Puromycin
Day 2
Day 4
Day 10
GFP/ Hoechst33342
c
ALB
CK19
Relative gene expression
Day 0
Day 10
TBG-GFP
Centrifugation+sorting
d
Donor 7 (TBG-GFP)
Donor 9 (TBG-GFP)
e
Doubling time (hour)
f
ALB
21.6%
CK19
99.9%
CD24
33.8%
CK18
99.9%
SOX9
20.4%
EPCAM
35.7%
HNF1A
99.9%
HNF4A
98.5%
Normalized To Mode

**Supplementary information, fig. S2 Lineage Tracing of PHCs, related to Fig. 1.** (a) Schematic representation of the TBG-EGFP-T2A-puro lentivirus vectors. (b) Primary liver cells were plated on a Matrigel-coated culture dish in hepatocyte maintenance medium (Lonza) overnight. Then, the cells were infected with a lentivirus carrying TBG-EGFP-T2A-puro for 24 hours and treated with puromycin for 2 days. Cells were then cultured in TEM for another 6 days. Red arrow indicates uninfected cell. Scale bars, 20 μm. (c) QPCR analyses for the expression of the indicated genes during TEM culture at day 0 and day 10, n = 4 technical replicates. (d) Light microscopy images of TBG-GFP labeled HepLPCs derived from 2 donors in TEM at passage 10. Scale bars, 100μm. (e) Doubling time calculated for 2 donors. Error bars represent s.d.; n = 3 technical replicates. (f) Flow cytometric analysis showing the proportion of ALB, CK18, HNF1A, CK19, SOX9, HNF4A, CD24 and EpCAM positive cells in TBG-GFP labeled liver cells (passage 5). Blue, positive cells; red, negative controls.
