# Supplementary material for: Expansion and differentiation of human hepatocyte-derived liver progenitor-like cells and their use for the study of hepatotropic pathogens
Source: Cell Res. 2018 Oct 25;29(1):8–22. doi: 10.1038/s41422-018-0103-x (PMC6318298; doi:10.1038/s41422-018-0103-x)
Supplement: Supplementary file 3 — Supplementary information, Figure S3 [file 41422_2018_103_MOESM3_ESM.pdf]

Fig. S3

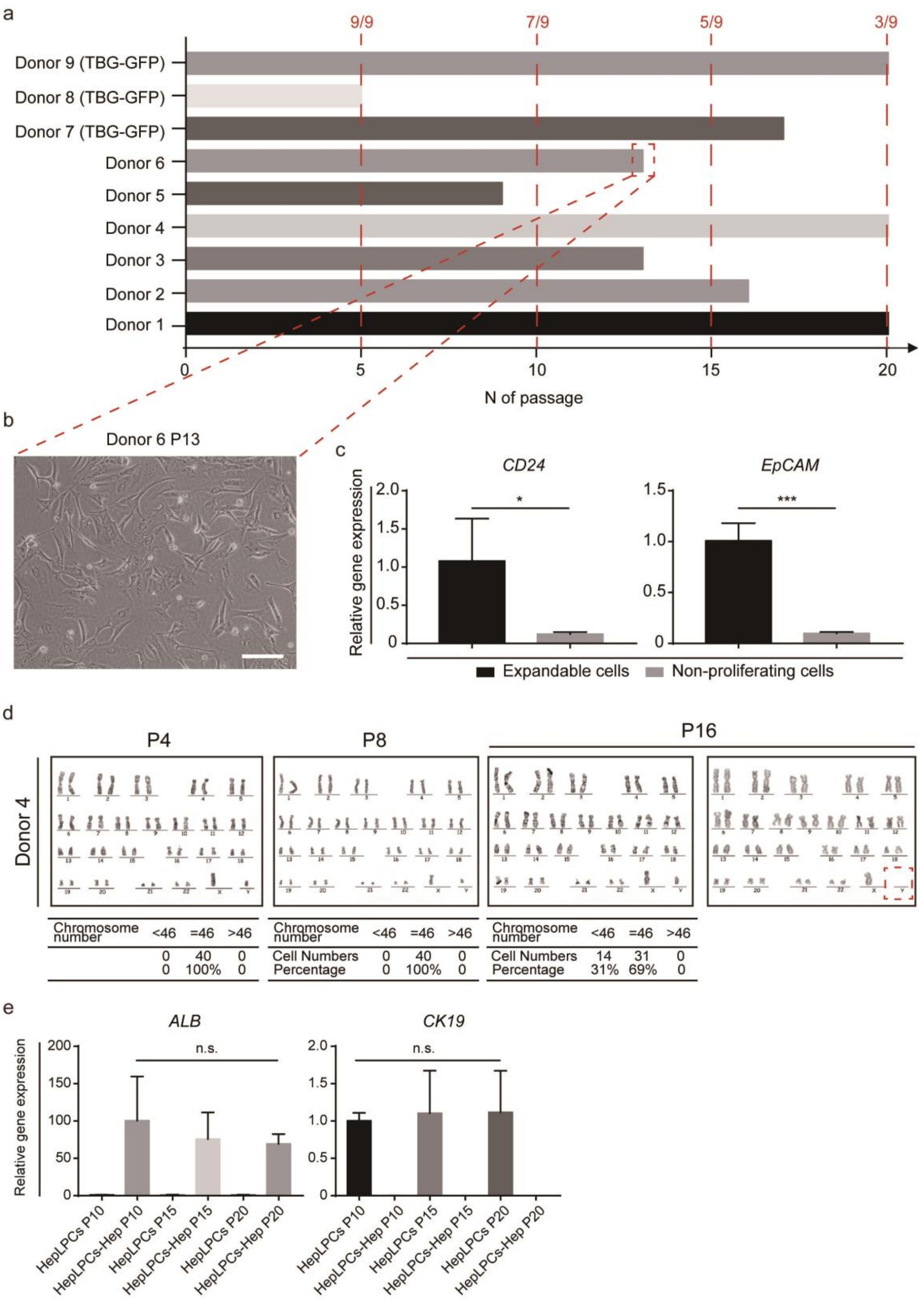
a
9/9
7/9
5/9
3/9
Donor 9 (TBG-GFP)
Donor 8 (TBG-GFP)
Donor 7 (TBG-GFP)
Donor 6
Donor 5
Donor 4
Donor 3
Donor 2
Donor 1
0
5
10
15
20
N of passage
b
Donor 6 P13
c
CD24
EpCAM
Relative gene expression
*
***
Expandable cells
Non-proliferating cells
d
P4
P8
P16
Donor 4
Chromosome number <46 =46 >46
0 40 0
0 100% 0
Chromosome number <46 =46 >46
Cell Numbers 0 40 0
Percentage 0 100% 0
Chromosome number <46 =46 >46
Cell Numbers 14 31 0
Percentage 31% 69% 0
e
ALB
CK19
Relative gene expression
n.s.
n.s.
HepLPCs P10
HepLPCs-Hep P10
HepLPCs P15
HepLPCs-Hep P15
HepLPCs P20
HepLPCs-Hep P20

Supplementary information, fig. S3 Individual variations in proliferative potential of HepLPCs from different donors, related to Fig. 1. (a) Expansion potential of HepLPCs. (b) Light microscopy images of HepLPCs derived from one donor in TEM at passage 13 with decreased cell proliferation capacity. Scale bar, 100μm. (c) QPCR analyses for the expression CD24 and EpCAM between expandable cells and non-proliferating cells. Error bars represent s.d. (* $p<0.05$, *** $p<0.001$, n=3). (d) Representative karyotype images of different passage of HepLPCs from donor 4. (e) QPCR analyses for the expression of CK19 and ALB in HepLPCs without or with differentiation at passage 10, passage 15 and passage 20. Error bars represent s.d. (n.s., non-significant, n=3).
