# Supplementary material for: Expansion and differentiation of human hepatocyte-derived liver progenitor-like cells and their use for the study of hepatotropic pathogens
Source: Cell Res. 2018 Oct 25;29(1):8–22. doi: 10.1038/s41422-018-0103-x (PMC6318298; doi:10.1038/s41422-018-0103-x)
Supplement: Supplementary file 4 — Supplementary information, Figure S4 [file 41422_2018_103_MOESM4_ESM.pdf]

Fig. S4

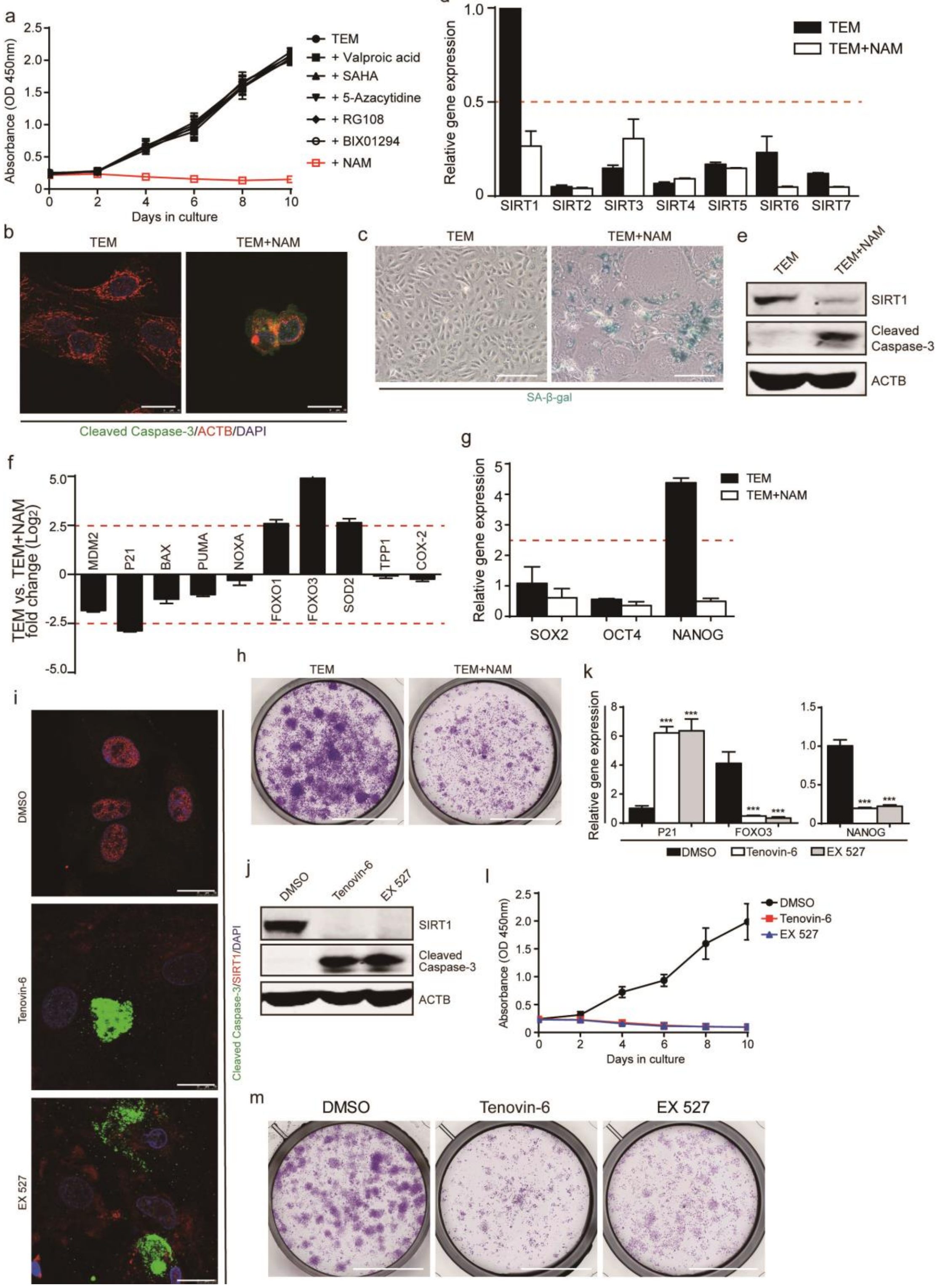
a
Absorbance (OD 450nm)
Days in culture
TEM
+ Valproic acid
+ SAHA
+ 5-Azacytidine
+ RG108
+ BIX01294
+ NAM
d
Relative gene expression
TEM
TEM+NAM
SIRT1 SIRT2 SIRT3 SIRT4 SIRT5 SIRT6 SIRT7
b
TEM
TEM+NAM
Cleaved Caspase-3/ACTB/DAPI
c
TEM
TEM+NAM
SA-β-gal
e
TEM
TEM+NAM
SIRT1
Cleaved Caspase-3
ACTB
f
TEM vs. TEM+NAM fold change (Log2)
MDM2 P21 BAX PUMA NOXA FOXO1 FOXO3 SOD2 TPP1 COX-2
g
Relative gene expression
TEM
TEM+NAM
SOX2 OCT4 NANOG
h
TEM
TEM+NAM
i
DMSO
Tenovin-6
EX 527
Cleaved Caspase-3/SIRT1/DAPI
k
Relative gene expression
P21 FOXO3 NANOG
DMSO
Tenovin-6
EX 527
j
DMSO
Tenovin-6
EX 527
SIRT1
Cleaved Caspase-3
ACTB
l
Absorbance (OD 450nm)
Days in culture
DMSO
Tenovin-6
EX 527
m
DMSO
Tenovin-6
EX 527

Supplementary information, fig. S4 Inhibition of SIRT1 signaling prevents the conversion, related to Fig. 2. (a) CCK-8 analyses of HepLPCs in TEM with epigenetic modifiers. 2 mM Valproic acid and 5 mM SAHA, HDAC inhibitors. 2 mM 5-Azacytidine and 0.04μM RG108, DNA methylation inhibitors. 1 μM BIX01294, H3K9 methyltransferase family inhibitor. 10 mM NAM, nicotinamide, sirtuin deacetylase family inhibitor. Error bars represent s.d.; n = 5 technical replicates from one donor. (b) Immunofluorescence images of Cleaved Caspase-3 and ACTB in PHCs cultured in TEM without (left) or with (right) NAM at day 4. Scale bars, 20μm. (c) PHCs cultured in TEM with NAM show sign of cell senescence (SA-β-gal staining) as compared to that in TEM at day 6. Scale bars, 100 μm. (d) QPCR analyses for the expression of sirtuin family genes of PHCs in TEM or TEM with NAM at day 4. Error bars represent s.d.; n = 3 donors. (e) Western blot analysis of SIRT1 and Cleaved Caspase-3 expression in TEM or TEM with NAM at day 4. ACTB as the loading control. (f and g) QPCR analyses for the expression of SIRT1-targeted genes (f) and SIRT1-related stemness genes (g) of PHCs in TEM or TEM with NAM at day 4. Error bars represent s.d.; n = 3 donors. (h) Light microscopy images demonstrating a reduction in clone formation in TEM with NAM versus TEM by crystal violet staining. Scale bars, 10mm. (i) Immunofluorescence images of SIRT1 and Cleaved Caspase-3 in PHCs in TEM, TEM with 5 μM Tenovin-6 and TEM with 50 μM EX 527 at day 4. Scale bars, 20μm. (j) Western blot analysis of SIRT1 and Cleaved Caspase-3 expression in TEM, TEM with Tenovin-6 and TEM with EX 527 at day 4. ACTB as the loading control. (k) QPCR analyses for the expression of P21, FOXO3 and NANOG in TEM, TEM with Tenovin-6 and TEM with EX 527 at day 4.

Error bars represent s.d.; n = 3 donors (one-way ANOVA with Dunnett correction for multiple comparisons, *** $p<0.001$). (l) CCK-8 analyses demonstrating suppression of cell proliferation in the presence of Tenovin-6 or EX 527 at day 10. Error bars represent s.d.; n = 5 technical replicates from one donor. (m) Light microscopy images show a reduction in clone formation following inhibition of SIRT1 with Tenovin-6 or EX 527 by crystal violet staining. Scale bars, 10mm.
