# Supplementary material for: Expansion and differentiation of human hepatocyte-derived liver progenitor-like cells and their use for the study of hepatotropic pathogens
Source: Cell Res. 2018 Oct 25;29(1):8–22. doi: 10.1038/s41422-018-0103-x (PMC6318298; doi:10.1038/s41422-018-0103-x)
Supplement: Supplementary file 5 — Supplementary information, Figure S5 [file 41422_2018_103_MOESM5_ESM.pdf]

Fig. S5

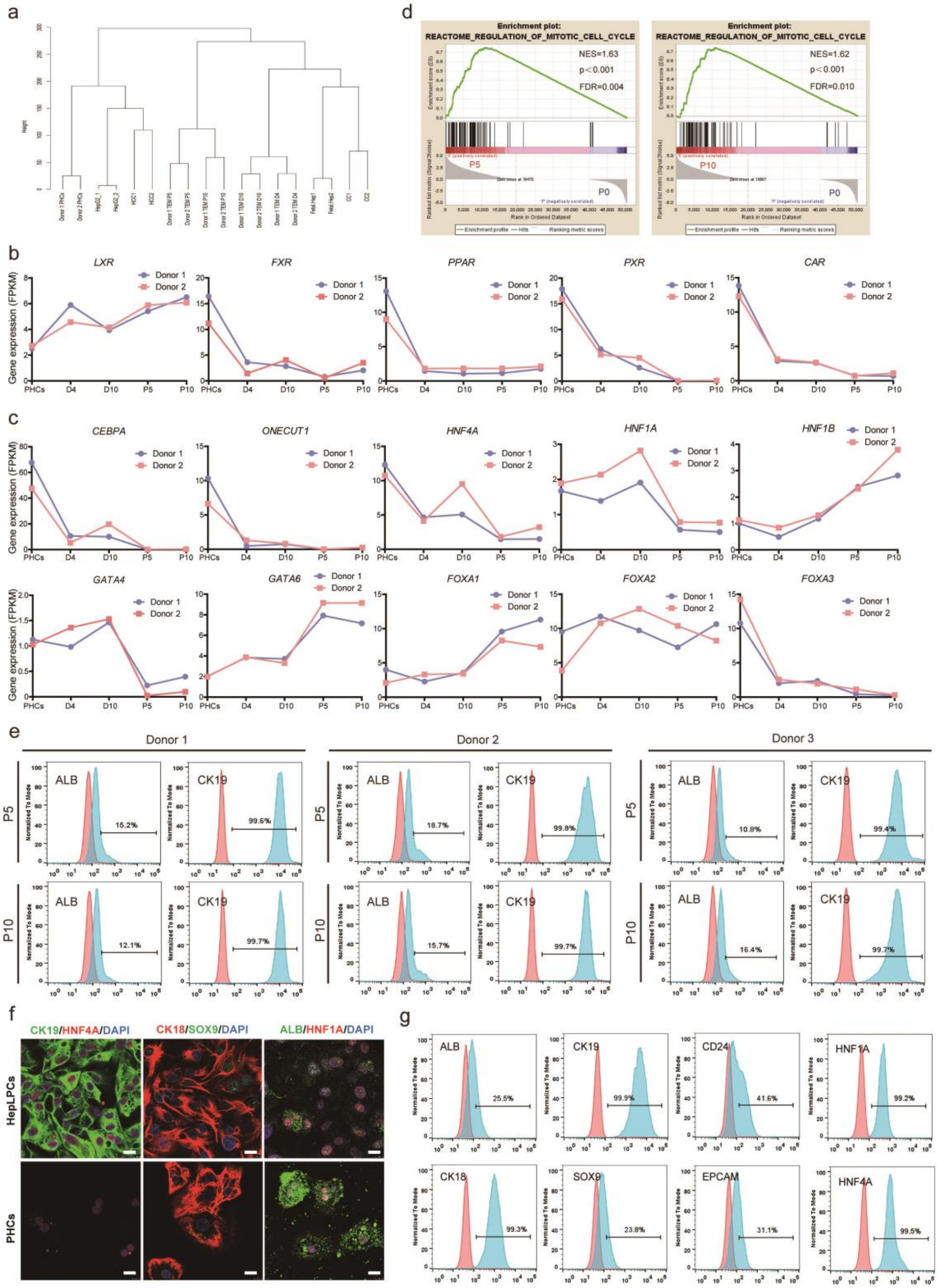
a
Height
Donor 1 PHCs
Donor 2 PHCs
HepG2_1
HepG2_2
HCC1
HCC2
Donor 1 TEM P5
Donor 2 TEM P5
Donor 1 TEM P10
Donor 2 TEM P10
Donor 1 TEM D10
Donor 2 TEM D10
Donor 1 TEM D4
Donor 2 TEM D4
Fetal Hep1
Fetal Hep2
CC1
CC2
d
Enrichment plot:
REACTOME_REGULATION_OF_MITOTIC_CELL_CYCLE
NES=1.63
p<0.001
FDR=0.004
P5
P0
NES=1.62
p<0.001
FDR=0.010
P10
Rank in Ordered Dataset
Enrichment profile
Hits
Ranking metric scores
b
Gene expression (FPKM)
LXR
FXR
PPAR
PXR
CAR
Donor 1
Donor 2
PHCs
D4
D10
P5
P10
c
CEBPA
ONECUT1
HNF4A
HNF1A
HNF1B
GATA4
GATA6
FOXA1
FOXA2
FOXA3
e
Donor 1
Donor 2
Donor 3
P5
P10
ALB
CK19
15.2%
99.6%
12.1%
99.7%
18.7%
99.8%
15.7%
99.7%
10.8%
99.4%
16.4%
99.7%
Normalized To Mode
f
CK19/HNF4A/DAPI
CK18/SOX9/DAPI
ALB/HNF1A/DAPI
HepLPCs
PHCs
g
ALB
25.5%
CK19
99.9%
CD24
41.6%
HNF1A
99.2%
CK18
99.3%
SOX9
23.8%
EPCAM
31.1%
HNF4A
99.5%

Supplementary information, fig. S5 Expression changes of hepatic markers during hepatocyte-to-LPC conversion and expansion, related to Fig. 3. (a) Hierarchical clustering based on global gene expression profile. (b) Gene expression of hepatic nuclear receptor family and transcription factor family (c) in PHCs and samples at day 4, day 10, passage 5 and passage 10 in TEM from 2 donors. (d) Gene Set Enrichment Analysis (GSEA) was performed to assess cell proliferation capacity by comparing passage 5 or passage 10 samples with PHCs. (e) Flow cytometric analysis showing the proportion of ALB or CK19 positive cells of HepLPCs at passage 5 and passage 10 derived from 3 individual donors. Blue, positive cells; red, negative controls. (f) Immunofluorescence images for the expression of representative markers in HepLPCs and PHCs (collected overnight after seeding as a monolayer). Scale bars, 20μm. (g) Flow cytometric analysis showing the proportion of ALB, CK18, HNF1A, CK19, SOX9, HNF4A, CD24 and EpCAM positive cells (passage 5) in the populations from donor 4. Blue, positive cells; red, negative controls.
