# Supplementary material for: Expansion and differentiation of human hepatocyte-derived liver progenitor-like cells and their use for the study of hepatotropic pathogens
Source: Cell Res. 2018 Oct 25;29(1):8–22. doi: 10.1038/s41422-018-0103-x (PMC6318298; doi:10.1038/s41422-018-0103-x)
Supplement: Supplementary file 6 — Supplementary information, Figure S6 [file 41422_2018_103_MOESM6_ESM.pdf]

Fig. S6

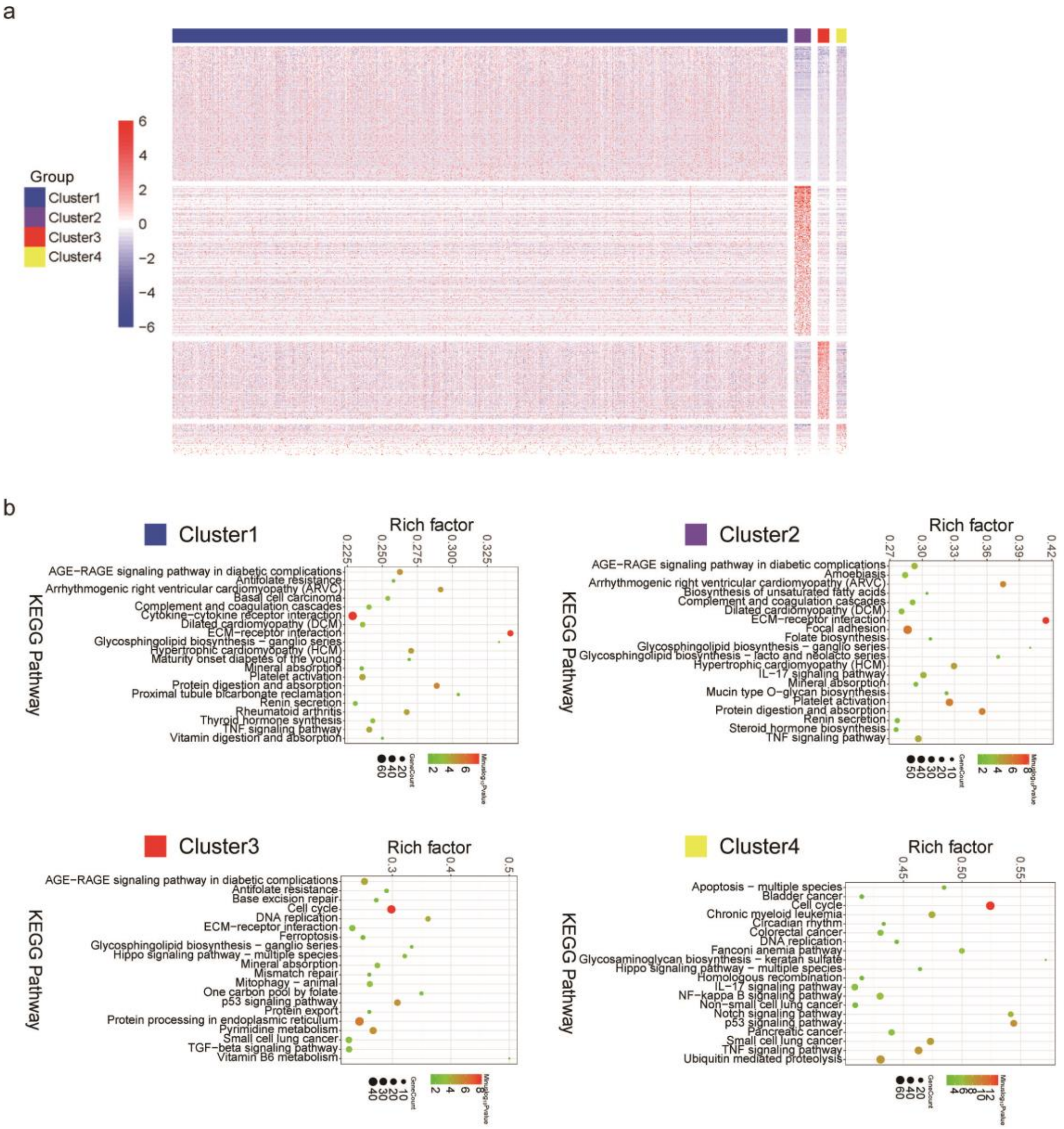
a
Group
Cluster1
Cluster2
Cluster3
Cluster4
b
Cluster1
Cluster2
Cluster3
Cluster4
Rich factor
KEGG Pathway
AGE−RAGE signaling pathway in diabetic complications
Antifolate resistance
Arrhythmogenic right ventricular cardiomyopathy (ARVC)
Basal cell carcinoma
Complement and coagulation cascades
Cytokine−cytokine receptor interaction
Dilated cardiomyopathy (DCM)
ECM−receptor interaction
Glycosphingolipid biosynthesis − ganglio series
Hypertrophic cardiomyopathy (HCM)
Maturity onset diabetes of the young
Mineral absorption
Platelet activation
Protein digestion and absorption
Proximal tubule bicarbonate reclamation
Renin secretion
Rheumatoid arthritis
Thyroid hormone synthesis
TNF signaling pathway
Vitamin digestion and absorption
Amoebiasis
Biosynthesis of unsaturated fatty acids
Focal adhesion
Folate biosynthesis
Glycosphingolipid biosynthesis − lacto and neolacto series
IL−17 signaling pathway
Mucin type O−glycan biosynthesis
Steroid hormone biosynthesis
Base excision repair
Cell cycle
DNA replication
Ferroptosis
Hippo signaling pathway − multiple species
Mismatch repair
Mitophagy − animal
One carbon pool by folate
p53 signaling pathway
Protein export
Protein processing in endoplasmic reticulum
Pyrimidine metabolism
Small cell lung cancer
TGF−beta signaling pathway
Vitamin B6 metabolism
Apoptosis − multiple species
Bladder cancer
Chronic myeloid leukemia
Circadian rhythm
Colorectal cancer
Fanconi anemia pathway
Glycosaminoglycan biosynthesis − keratan sulfate
Homologous recombination
NF−kappa B signaling pathway
Non−small cell lung cancer
Notch signaling pathway
Pancreatic cancer
Ubiquitin mediated proteolysis
GeneCount
MinusLog₁₀Pvalue

Supplementary information, fig. S6 Subgroup specific genes heatmap (a) and KEGG pathway enrichment analysis of corresponding gene groups (b), related to fig. 3.
