# Supplementary material for: Expansion and differentiation of human hepatocyte-derived liver progenitor-like cells and their use for the study of hepatotropic pathogens
Source: Cell Res. 2018 Oct 25;29(1):8–22. doi: 10.1038/s41422-018-0103-x (PMC6318298; doi:10.1038/s41422-018-0103-x)
Supplement: Supplementary file 7 — Supplementary information, Figure S7 [file 41422_2018_103_MOESM7_ESM.pdf]

Fig. S7

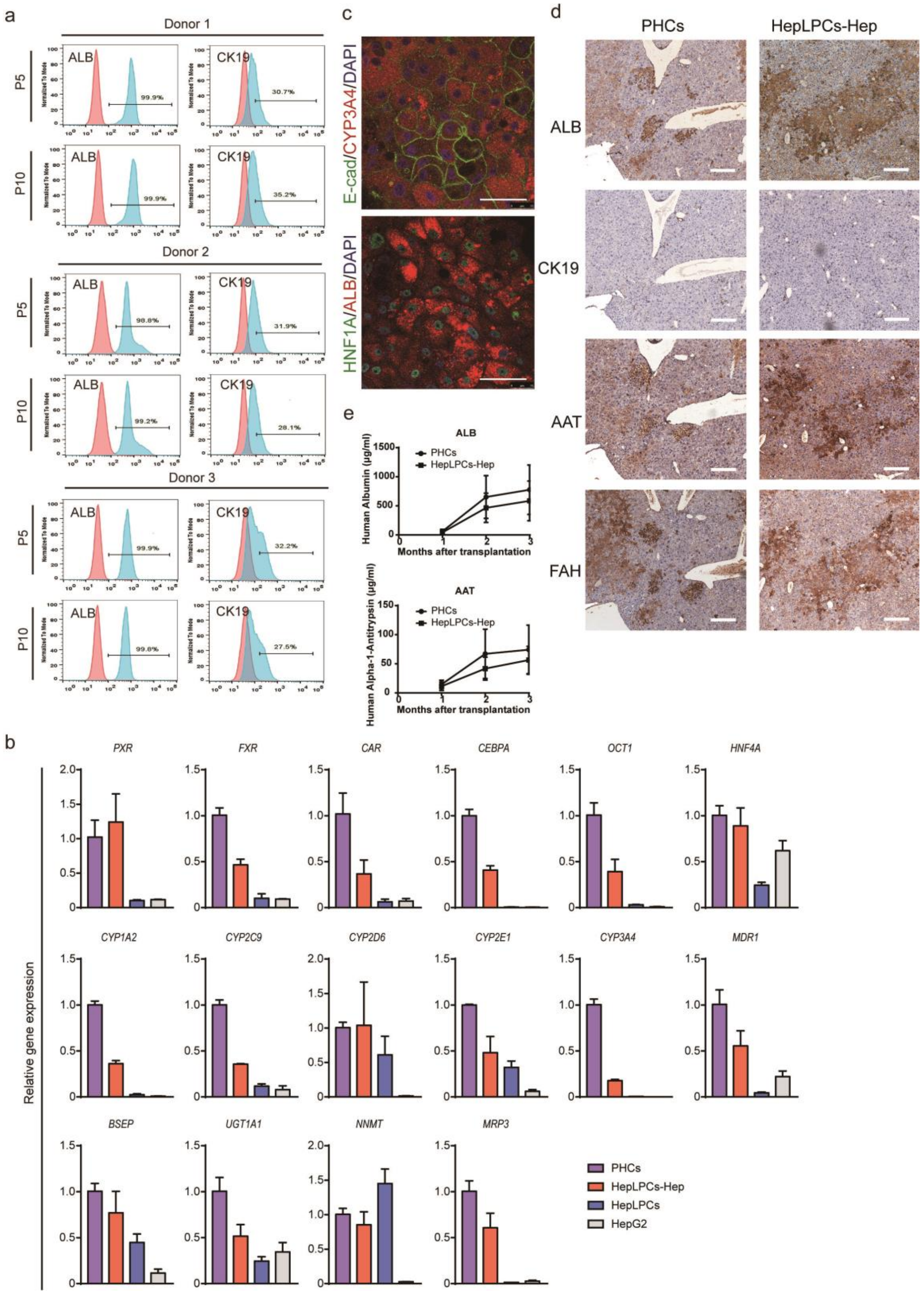
a
Donor 1
P5
P10
ALB
CK19
99.9%
30.7%
99.9%
35.2%
Donor 2
98.8%
31.9%
99.2%
28.1%
Donor 3
99.9%
32.2%
99.8%
27.5%
Normalized To Mode
c
E-cad/CYP3A4/DAPI
HNF1A/ALB/DAPI
d
PHCs
HepLPCs-Hep
ALB
CK19
AAT
FAH
e
ALB
Human Albumin (µg/ml)
Months after transplantation
AAT
Human Alpha-1-Antitrypsin (µg/ml)
PHCs
HepLPCs-Hep
b
PXR
FXR
CAR
CEBPA
OCT1
HNF4A
CYP1A2
CYP2C9
CYP2D6
CYP2E1
CYP3A4
MDR1
BSEP
UGT1A1
NNMT
MRP3
Relative gene expression
PHCs
HepLPCs-Hep
HepLPCs
HepG2

Supplementary information, fig. S7 Expression changes of hepatic markers during hepatic differentiation of HepLPCs, related to fig. 4. (a) Flow cytometric analysis showing the proportion of ALB or CK19 positive cells in HepLPCs-Hep differentiated from HepLPCs at passage 5 or 10 from 3 individual donors. Blue, positive cells; red, negative controls. (b) QPCR analyses for the expression of liver function related genes in PHCs (freshly isolated), HepLPCs-Hep, HepLPCs and HepG2. Error bars represent s.d.; n = 3 donors. (c) Immunofluorescence images of E-cadherin, CYP3A4, HNF1A and ALB in HepLPCs-Hep. Scale bars, 50μm. (d) Immunohistochemical staining of human ALB, CK19, AAT and FAH. Scale bars, 100μm. (e) Serum levels of human serum albumin and alpha-1-antitrypsin in mice transplanted with HepLPCs.
