# Supplementary material for: Expansion and differentiation of human hepatocyte-derived liver progenitor-like cells and their use for the study of hepatotropic pathogens
Source: Cell Res. 2018 Oct 25;29(1):8–22. doi: 10.1038/s41422-018-0103-x (PMC6318298; doi:10.1038/s41422-018-0103-x)
Supplement: Supplementary file 8 — Supplementary information, Figure S8 [file 41422_2018_103_MOESM8_ESM.pdf]

Fig. S8

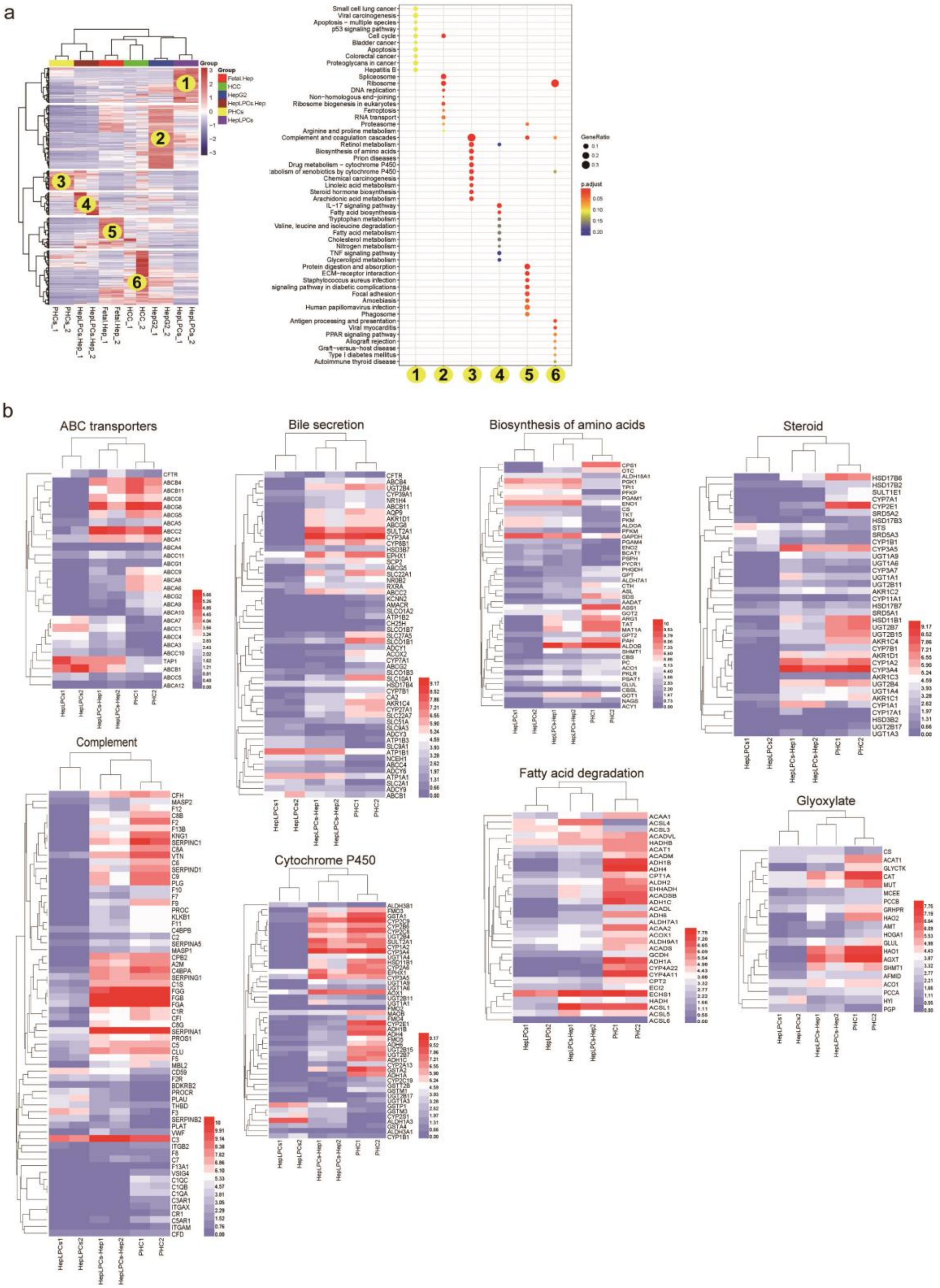
a
b
ABC transporters
Bile secretion
Biosynthesis of amino acids
Steroid
Complement
Cytochrome P450
Fatty acid degradation
Glyoxylate

Supplementary information, fig. S8 Heat maps for the expression of hepatic function-related genes, related to fig. 4. (a) Differential gene groups in euclidean hierarchical clustering (left) and KEGG pathway enrichment analysis of the corresponding region (right). (b) Euclidean hierarchical clustering for the expression of hepatic function-related genes by HepLPCs, HepLPCs-Hep and PHCs. Each element represents the $\log_2$ (normalized expression), as scaled by the corresponding color legends from 2 donors.
