# Supplementary material for: Expansion and differentiation of human hepatocyte-derived liver progenitor-like cells and their use for the study of hepatotropic pathogens
Source: Cell Res. 2018 Oct 25;29(1):8–22. doi: 10.1038/s41422-018-0103-x (PMC6318298; doi:10.1038/s41422-018-0103-x)
Supplement: Supplementary file 9 — Supplementary information, Figure S9 [file 41422_2018_103_MOESM9_ESM.pdf]

Fig. S9

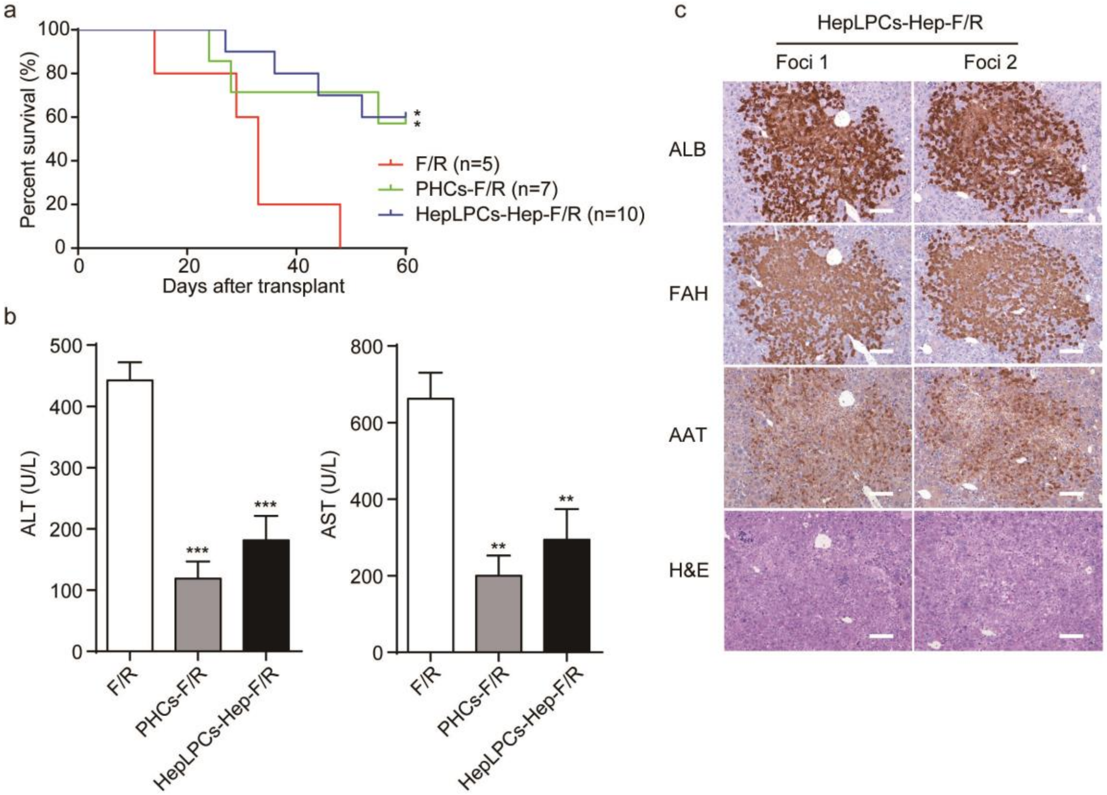
a
Percent survival (%)
100
80
60
40
20
0
0
20
40
60
Days after transplant
F/R (n=5)
PHCs-F/R (n=7)
HepLPCs-Hep-F/R (n=10)
*
*
b
ALT (U/L)
500
400
300
200
100
0
***
***
F/R
PHCs-F/R
HepLPCs-Hep-F/R
AST (U/L)
800
600
400
200
0
**
**
F/R
PHCs-F/R
HepLPCs-Hep-F/R
c
HepLPCs-Hep-F/R
Foci 1
Foci 2
ALB
FAH
AAT
H&E

Supplementary information, fig. S9 Survival studies, related to fig. 4. (a) Kaplan-Meier survival curve of F/R mice that received no cells, PHCs or HepLPCs-Hep after NTBC withdrawal. (b) Serum levels of ALT and AST in moribund control F/R mice (n = 4), surviving PHCs-F/R mice (n = 4), and surviving HepLPCs-Hep-F/R mice (n = 6). (c) The integration of HepLPCs-Hep cells in F/R livers was determined by H&E and immunostaining for human ALB, FAH and AAT in serial sections. Scale bars, 100μm.
