# Supplementary material for: Expansion and differentiation of human hepatocyte-derived liver progenitor-like cells and their use for the study of hepatotropic pathogens
Source: Cell Res. 2018 Oct 25;29(1):8–22. doi: 10.1038/s41422-018-0103-x (PMC6318298; doi:10.1038/s41422-018-0103-x)
Supplement: Supplementary file 10 — Supplementary information, Figure S10 [file 41422_2018_103_MOESM10_ESM.pdf]

Fig. S10

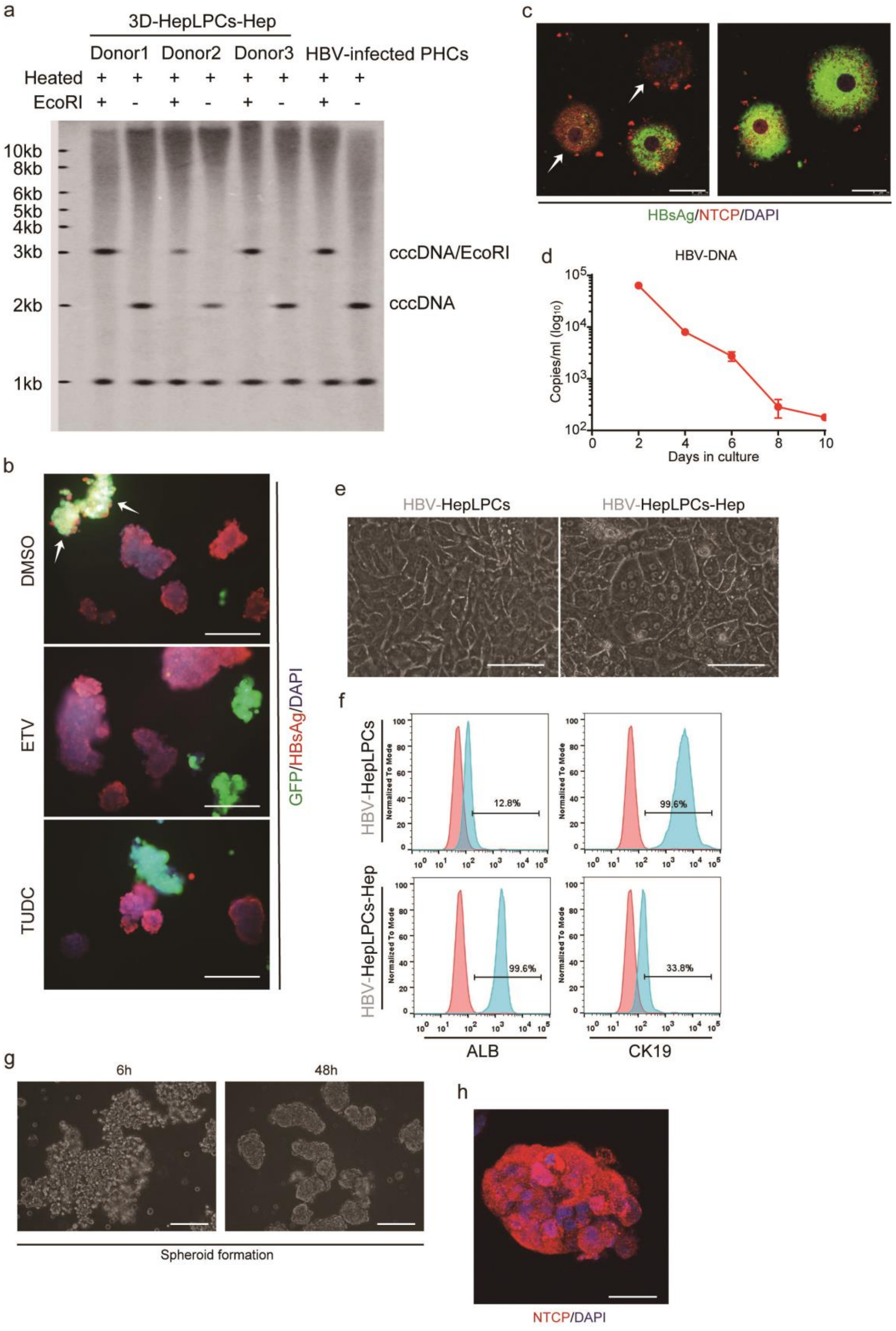
a
3D-HepLPCs-Hep
Donor1 Donor2 Donor3 HBV-infected PHCs
Heated + + + + + + + +
EcoRI + - + - + - + -
10kb
8kb
6kb
5kb
4kb
3kb
2kb
1kb
cccDNA/EcoRI
cccDNA
b
DMSO
ETV
TUDC
GFP/HBsAg/DAPI
c
HBsAg/NTCP/DAPI
d
HBV-DNA
Copies/ml (log10)
Days in culture
e
HBV-HepLPCs
HBV-HepLPCs-Hep
f
HBV-HepLPCs
HBV-HepLPCs-Hep
Normalized To Mode
12.8%
99.6%
99.6%
33.8%
ALB
CK19
g
6h
48h
Spheroid formation
h
NTCP/DAPI

Supplementary information, fig. S10 Characteristics of HepLPCs derived from HBV-infected patient and 3D spheroid differentiation, related to fig. 6. (a) Southern blot analysis for HBV cccDNA. (b) 3D-Diff HepLPCs were infected with HBV for 8 days, and then co-cultured with TBG-GFP labeled 3D-Diff HepLPCs with or without ETV or TUDC. Eight days after co-culture, HBsAg staining was used to evaluate HBV replication. Scale bar=200μm. (c) Immunofluorescence images of NTCP and HBsAg in PHCs derived from HBV-infected donors at day 0. Two representative fields are shown. Scale bars, 20μm. (d) Extracellular HBV-DNA in cells derived from HBV-infected livers cultured in TEM over 10 days. (e) Light microscopy images of HepLPCs and HepLPCs-Hep derived from HBV-infected donors. Scale bars, 50μm. (f) Flow cytometric analysis showing the proportion of ALB and CK19 positive cells in HepLPCs or HepLPCs-Hep from HBV-infected donor 3 passage 5. Blue, positive cells; red, negative controls. (g) Time course of cellular spheroid formation. Scale bars, 200 μm. (h) Immunofluorescence images of NTCP in 3D spheroid of patient-derived HepLPCs-Hep at day 15. Scale bar, 25μm.
