# Supplementary material for: Expansion and differentiation of human hepatocyte-derived liver progenitor-like cells and their use for the study of hepatotropic pathogens
Source: Cell Res. 2018 Oct 25;29(1):8–22. doi: 10.1038/s41422-018-0103-x (PMC6318298; doi:10.1038/s41422-018-0103-x)
Supplement: Supplementary file 11 — Supplementary information, Figure S11 [file 41422_2018_103_MOESM11_ESM.pdf]

Fig. S11

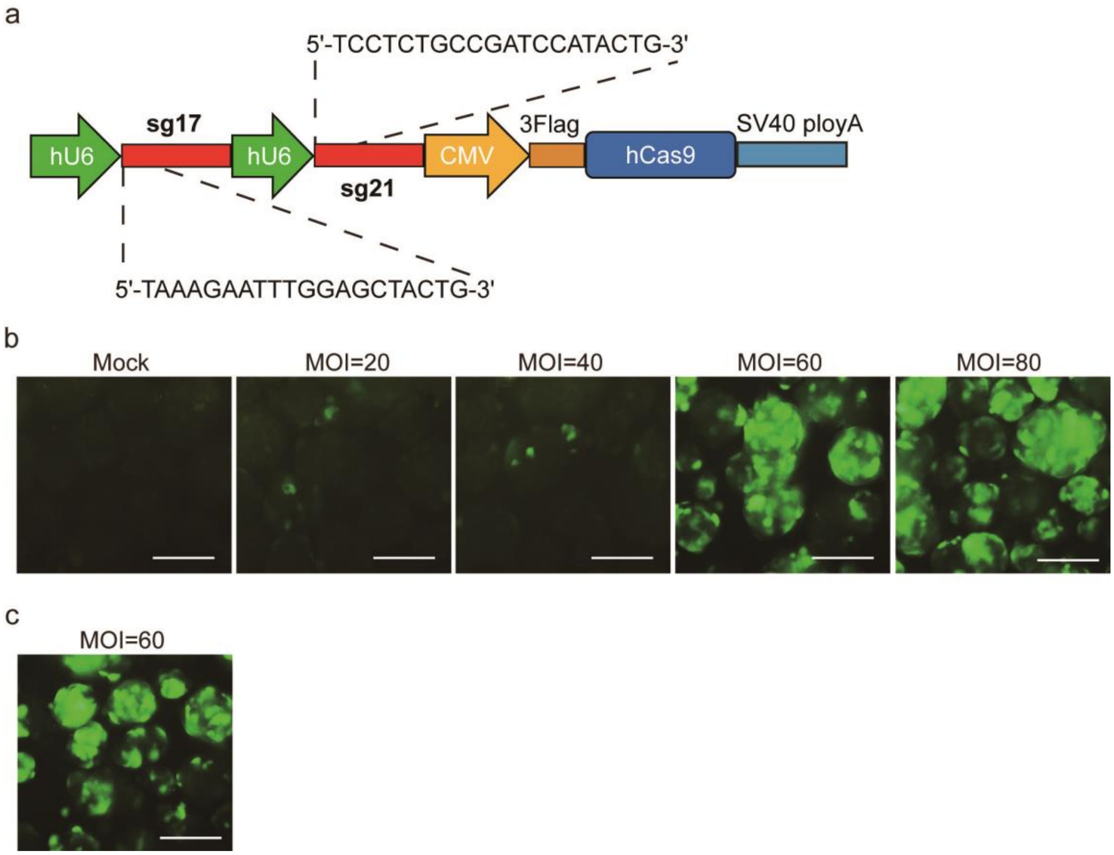
a
5'-TCCTCTGCCGATCCATACTG-3'
hU6
sg17
hU6
sg21
CMV
3Flag
hCas9
SV40 ployA
5'-TAAAGAATTTGGAGCTACTG-3'
b
Mock
MOI=20
MOI=40
MOI=60
MOI=80
c
MOI=60

Supplementary information, fig. S11 Adenoviral expression of Cas9/sgRNAs targeting HBV cccDNA, related to fig. 7. (a) Schematic diagram of adenoviral vector with HBV-sgRNAs CRISPR/Cas9 expression. (b) 3D-HepLPCs-Hep infected with CAS9/HBV at a MOI of 0, 20, 40, 60 or 80 within 48 hours. GFP, green; Scale bars, 200 μm. (c) GFP positive cells in 3D-HepLPCs-Hep infected with CAS9/HBV at a MOI of 60 were still observed at day 15. Scale bar, 200 μm.
