# Supplementary material for: Expansion and differentiation of human hepatocyte-derived liver progenitor-like cells and their use for the study of hepatotropic pathogens
Source: Cell Res. 2018 Oct 25;29(1):8–22. doi: 10.1038/s41422-018-0103-x (PMC6318298; doi:10.1038/s41422-018-0103-x)
Supplement: Supplementary file 12 — Supplementary information, Table S1 [file 41422_2018_103_MOESM12_ESM.pdf]

Supplementary information, Table S1 Donor information.

Identifier	Gender	Age	Associated pathology
Donor 1	Female	57	hepatic hemangioma
Donor 2	Female	45	hepatic hemangioma
Donor 3	Female	48	hepatic hemangioma
Donor 4	Male	39	hepatic hemangioma
Donor 5	Female	51	hepatic hemangioma
Donor 6	Female	42	hepatic hemangioma
Donor 7	Female	38	hepatic hemangioma
Donor 8	Male	49	hepatic hemangioma
Donor 9	Female	55	hepatic hemangioma
HBV-infected Donor 1	Female	53	hepatic hemangioma & chronic hepatitis B
HBV-infected Donor 2	Female	43	hepatic hemangioma & chronic hepatitis B
HBV-infected Donor 3	Male	59	hepatic hemangioma & chronic hepatitis B
