# Supplementary material for: Expansion and differentiation of human hepatocyte-derived liver progenitor-like cells and their use for the study of hepatotropic pathogens
Source: Cell Res. 2018 Oct 25;29(1):8–22. doi: 10.1038/s41422-018-0103-x (PMC6318298; doi:10.1038/s41422-018-0103-x)
Supplement: Supplementary file 13 — Supplementary information, Table S2 [file 41422_2018_103_MOESM13_ESM.pdf]

Supplementary information, Table S2 Antibody list.

Primary antibody list					
**Antibody**	**Company**	**Product Codes**	**Ig Species**	**Conjugate**	**Dilution**
EpCAM	Biolegend	324204	Mouse IgG2b	FITC	1:50 (FC)
Isotype Ctrl	Biolegend	400310	Mouse IgG2b	FITC	1:50 (FC)
CD24	Biolegend	311108	Mouse IgG2a	Alexa Fluor® 488	1:50 (FC)
Isotype Ctrl	Biolegend	400233	Mouse IgG2a	Alexa Fluor® 488	1:50 (FC)
ASGPR1	Bdbiosciences	563655	Mouse IgG1	PE	1:50 (FC)
ALB	Bethyl	A80-229A	Goat IgG	none	1:100(IHC)
ALB	Abcam	ab207327	Rabbit IgG	none	1:20 (FC), 1:100(IF)
CK19	Abcam	ab76539	Rabbit IgG	none	1:20 (FC), 1:100(IF), 1:100(IHC)
Isotype Ctrl	Cell Signaling Technology	2975	Rabbit IgG	Alexa Fluor® 488	1:50 (FC)
CK18	Abcam	ab668	Mouse IgG1	none	1:50 (FC), 1:100(IF)
Isotype Ctrl	Cell Signaling Technology	4878	Mouse IgG1	Alexa Fluor® 488	1:50 (FC)
SOX9	Abcam	ab185966	Rabbit IgG	none	1:50 (FC), 1:100(IF)
HNF1A	Santa Cruz	sc-393925	Mouse IgG2a	none	1:50 (FC), 1:100(IF)
HNF4A	Abcam	ab201460	Rabbit IgG	none	1:500 (FC), 1:1000(IF)
HBsAg	Abcam	ab68520	Rabbit IgG	none	1:100(IF)
AFP	Abcam	ab46799	Rabbit IgG	none	1:100(IHC)
KI67	Abcam	ab15580	Rabbit IgG	none	1:100(IHC)
E-cad	Abcam	ab1416	Mouse IgG1	none	1:100(IF)
CYP3A4	Proteintech	18227	Rabbit IgG	none	1:50(IF)
NTCP	Aviva Biosystems	OABF00881	Rabbit IgG	none	1:50(IF), 1:500(WB)
SIRT1	Proteintech	60303	Mouse IgG2b	none	1:100(IF), 1:1000(WB)
Cleaved Caspase-3	Cell Signaling Technology	9661	Rabbit IgG	none	1:400(IF), 1:1000(WB)
ACTB	Cell Signaling Technology	3700	Mouse IgG2b	none	1:1000(IF), 1:1000(WB)
GAPDH	Cell Signaling Technology	97166	Mouse IgG1	none	1:1000(WB)

Secondary antibody list			
**Antibody**	**Company**	**Product Codes**	**Dilution**
Goat anti-Mouse IgG (H+L), Alexa Fluor 488	Invitrogen	A-11001	1:200
Goat anti-Rabbit IgG (H+L), Alexa Fluor 488	Invitrogen	A-11034	1:200
Goat anti-Mouse IgG (H+L), Alexa Fluor 555	Invitrogen	A-21422	1:200
Goat anti-Rabbit IgG (H+L), Alexa Fluor 555	Invitrogen	A-21428	1:200
Goat anti-Mouse IgG (H+L), HRP	Invitrogen	31430	1:5000
Goat anti-Rabbit IgG (H+L), HRP	Invitrogen	31460	1:5000
Rabbit anti-Goat IgG (H+L), HRP	Invitrogen	61-1620	1:5000
