# Supplementary material for: Expansion and differentiation of human hepatocyte-derived liver progenitor-like cells and their use for the study of hepatotropic pathogens
Source: Cell Res. 2018 Oct 25;29(1):8–22. doi: 10.1038/s41422-018-0103-x (PMC6318298; doi:10.1038/s41422-018-0103-x)
Supplement: Supplementary file 14 — Supplementary information, Table S3 [file 41422_2018_103_MOESM14_ESM.pdf]

Supplementary information, Table S3 Primer list.

Gene	Forward sequence 5' -> 3'	Reverse sequence 5' -> 3'
ALB	GAGACCAGAGGTTGATGTGATG	AGTTCCGGGGCATAAAAGTAAG
G6PC	GTGTCCGTGATCGCAGACC	GACGAGGTTGAGCCAGTCTC
HNF4A	GATGTAGTCCTCCAAGCTCAC	GCCATCATCTTCTTTGACCCA
CK19	AACGGCGAGCTAGAGGTGA	GGATGGTCGTGTAGTAGTGGC
CK7	TCCGCGAGGTCACCATTAAC	GCTCTGTCAACTCCGTCTCAT
SOX9	AGCGAACGCACATCAAGAC	CTGTAGGCGATCTGTTGGGG
P21	CGATGGAACTTCGACTTTGTCA	GCACAAGGGTACAAGACAGTG
FOXO3	TCACGCACCAATTCTAACGC	CACGGCTTGCTTACTGAAGG
NANOG	TTTGTGGGCCTGAAGAAAACT	AGGGCTGTCCTGAATAAGCAG
CK18	GTTGACCGTGGAGGTAGATGC	GAGCCAGCTCGTCATATTGGG
CD24	CTCCTACCCACGCAGATTTATTC	AGAGTGAGACCACGAAGAGAC
EpCAM	AATCGTCAATGCCAGTGTACTT	TCTCATCGCAGTCAGGATCATAA
AFP	CTTTGGGCTGCTCGCTATGA	GCATGTTGATTTAACAAGCTGCT
CYP1A2	CTGGGCACTTCGACCCTTAC	TCTCATCGCTACTCTCAGGGA
CYP2B6	CCGGGGATATGGTGTGATCTT	CCGAAGTCCCTCATAGTGGTC
CYP3A4	GTGGGGCTTTTATGATGGTCA	ACATCTCCATACTGGGCAATGA
cccDNA	GCCTATTGATTGGAAAGTATGT	AGCTGAGGCGGTATCTA
3.5kb RNA	CTCCTCCAGCTTATAGACC	GTGAGTGGGCCTACAAA
SIRT1	TAGCCTTGTCAGATAAGGAAGGA	ACAGCTTCACAGTCAACTTTGT
SIRT2	TGCGGAACTTATTCTCCCAGA	GAGAGCGAAAGTCGGGGAT
SIRT3	ACCCAGTGGCATTCCAGAC	GGCTTGGGGTTGTGAAAGAAG
SIRT4	GCTTTGCGTTGACTTTCAGGT	CCAATGGAGGCTTTCGAGCA
SIRT5	GCCATAGCCGAGTGTGAGAC	CAACTCCACAAGAGGTACATCG
SIRT6	CCCACGGAGTCTGGACCAT	CTCTGCCAGTTTGTCCCTG
SIRT7	AGAAGCGTTAGTGCTGCCG	GAGCCCGTCACAGTTCTGAG
SOX2	TACAGCATGTCCTACTCGCAG	GAGGAAGAGGTAACCACAGGG
OCT4	CTTGAATCCCGAATGGAAAGGG	GTGTATATCCCAGGGTGATCCTC
MDM2	GAATCATCGGACTCAGGTACATC	TCTGTCTCACTAATTGCTCTCCT
BAX	CCCGAGAGGTCTTTTTCCGAG	CCAGCCCATGATGGTTCTGAT
PUMA	GCCAGATTTGTGAGACAAGAGG	CAGGCACCTAATTGGGCTC
NOXA	ACCAAGCCGGATTTGCGATT	ACCAAGCCGGATTTGCGATT
FOXO1	GGATGTGCATTCTATGGTGTACC	TTTCGGGATTGCTTATCTCAGAC
FOXO3	CGGACAAACGGCTCACTCT	GGACCCGCATGAATCGACTAT
SOD2	TTTCAATAAGGAACGGGGACAC	GTGCTCCCACACATCAATCC
TPP1	GTTTCATCACTATGTGGGAGGAC	GTATCGCTTACGGATCACAGAG
COX-2	CTGGCGCTCAGCCATACAG	CGCACTTATACTGGTCAAATCCC
ACTB	GCCTCGCTGTCCACCTTCC	TGCTGTCACCTTCACCGTTCC
PXR	TTGCCCATCGAGGACCAGAT	GTCTCCGCGTTGAACACTGT
FXR	TGCAGATCAGACCGTGAATGA	TTGGTTGCCATTTCCGTCAAA
CAR	GATGCTGGCATGAGGAAAGAC	TTGCTCCTTACTCAGTTGCAC
CEBPA	TATAGGCTGGGCTTCCCCTT	AGCTTTCTGGTGTGACTCGG
OCT1	GAACATGGGAAGGATAGAGGCA	GTAGAGTTCGACGCTGGACAT
CYP2C9	GCCTGAAACCCATAGTGGTG	GGGGCTGCTCAAAATCTTGATG
CYP2D6	TGGCAAGGTCCTACGCTTC	GCCACCACTATGCACAGGTT
CYP2E1	ATGTCTGCCCTCGGAGTCA	CGATGATGGGAAGCGGGAAA
MDR1	GGGAGCTTAACACCCGACTTA	GCCAAAATCACAAGGGTTAGCTT
BSEP	TTGGCTGATGTTTGTGGGAAG	CCAAAAATGAGTAGCACGCCT
UGT1A1	TTGTCTGGCTGTTCCCACTTA	GGTCCGTCAGCATGACATCA
NNMT	ATATTCTGCCTAGACGGTGTGA	TCAGTGACGACGATCTCCTTAAA
MRP3	ATTCCACTCAACGGAGCTGTG	GCGCGAGTCCTTCAATTTCAT
RXRA	GGAGGTGAGGGAGGAGTT	GCATGAGTTAGTCGCAGACAT
NTCP	TGCTCTTCCCCACATTGATG	TCCTGGTTCTCATTCCTTGC
