# Supplementary material for: Expansion and differentiation of human hepatocyte-derived liver progenitor-like cells and their use for the study of hepatotropic pathogens
Source: Cell Res. 2018 Oct 25;29(1):8–22. doi: 10.1038/s41422-018-0103-x (PMC6318298; doi:10.1038/s41422-018-0103-x)
Supplement: Supplementary file 19 — Supplementary movie legend [file 41422_2018_103_MOESM19_ESM.docx]

**Supplementary information, Movie S1 PHCs cultured in HGM from day 2 to day 8 as shown by time-lapse imaging.**

**Supplementary information, Movie S2 PHCs cultured in TEM from day 2 to day 8 as shown by time-lapse imaging.**

**Supplementary information, Movie S3 HBsAg staining in 3D-HepLPCs without differentiation (3D-Diff Day 0) at 8 days post infection as shown by Leica TCS SP8 Z-Stack.**

**Supplementary information, Movie S4 HBsAg staining in 3D-HepLPCs with differentiation (3D-Diff Day 10) at 8 days post infection as shown by Leica TCS SP8 Z-Stack.**
